# Is qualitative social research in global health fulfilling its potential?: a systematic evidence mapping of research on point-of-care testing in low- and middle-income contexts

**DOI:** 10.1186/s12913-024-10645-5

**Published:** 2024-02-07

**Authors:** Janet Perkins, Sarah Nelson, Emma Birley, Emilie Mcswiggan, Marshall Dozier, Anna McCarthy, Nadege Atkins, Eldad Agyei-Manu, Jasmin Rostron, Koichi Kameda, Ann Kelly, Clare Chandler, Alice Street

**Affiliations:** 1 Department of Social Anthropology, School of Social and Political Science, University of Edinburgh, Chrystal Macmillan Building, 15a George Square, Edinburgh, EH8 9LD Scotland, UK; 2 Centre for Population Health Sciences, Old Medical School, Usher Institute, University of Edinburgh, Teviot Place, Edinburgh, EH8 9AG Scotland, UK; 3 Library Academic Support Team, Library & University Collections, and Information Services University of Edinburgh, Argyle House, 3 Lady Lawson Street, Edinburgh, EH3 9DR Scotland, UK; 4 Department of Social Work, School of Social and Political Science, University of Edinburgh, Chrystal Macmillan Building, 15a George Square, Edinburgh, EH8 9LD Scotland, UK; 5 Centre Population et Développement (CEPED), 45 Rue Des Saints-Pères, 75006 Paris, France; 6 Department of Global Health and Social Medicine, King’s College London, Bush House North East Wing, 30 Aldwych, London, WC2B 4BG England, UK; 7 Department of Global Health and Development, London School of Hygiene and Tropical Medicine, 15-17 Tavistock Place, London, WC1H 9SH England, UK

**Keywords:** Point-of-care testing, Diagnosis, Qualitative research, Social sciences, Developing countries, Global health, Low- and middle-income countries, Systematic evidence mapping, Decolonisation

## Abstract

**Background:**

Qualitative social research has made valuable contributions to understanding technology-based interventions in global health. However, we have little evidence of who is carrying out this research, where, how, for what purpose, or the overall scope of this body of work. To address these questions, we undertook a systematic evidence mapping of one area of technology-focused research in global health, related to the development, deployment and use of point-of-care tests (POCTs) for low-and middle-income countries (LMICs).

**Methods:**

We conducted an exhaustive search to identify papers reporting on primary qualitative studies that explore the development, deployment, and use of POCTs in LMICs and screened results to identify studies meeting the inclusion criteria. Data were extracted from included studies and descriptive analyses were conducted.

**Results:**

One hundred thirty-eight studies met our inclusion criteria, with numbers increasing year by year. Funding of studies was primarily credited to high income country (HIC)-based institutions (95%) and 64% of first authors were affiliated with HIC-based institutions. Study sites, in contrast, were concentrated in a small number of LMICs. Relatively few studies examined social phenomena related to POCTs that take place in HICs. Seventy-one percent of papers reported on studies conducted within the context of a trial or intervention. Eighty percent reported on studies considering POCTs for HIV and/or malaria. Studies overwhelmingly reported on POCT use (91%) within primary-level health facilities (60%) or in hospitals (30%) and explored the perspectives of the health workforce (70%).

**Conclusions:**

A reflexive approach to the role, status, and contribution of qualitative and social science research is crucial to identifying the contributions it can make to the production of global health knowledge and understanding the roles technology can play in achieving global health goals. The body of qualitative social research on POCTs for LMICs is highly concentrated in scope, overwhelmingly focuses on testing in the context of a narrow number of donor-supported initiatives and is driven by HIC resources and expertise. To optimise the full potential of qualitative social research requires the promotion of open and just research ecosystems that broaden the scope of inquiry beyond established public health paradigms and build social science capacity in LMICs.

**Supplementary Information:**

The online version contains supplementary material available at 10.1186/s12913-024-10645-5.

## Background

The era of ‘global health’ has been characterised by a growing emphasis on technical solutions to what are ultimately problems of health inequities [1, 2]. In recent years, mobile, easy-to-use and affordable technological innovations, such as pharmaceuticals, m-health devices, and rapid diagnostic tests, have become increasingly central to efforts to improve access to life-saving prevention, diagnosis, and treatment in places where existing health systems and infrastructures are under-resourced [1–5].

Qualitative social research has made widely recognised contributions to understanding the implications and effects of this innovation-focused agenda, especially in terms of the dynamic interrelationships between technologies, infrastructures, health systems, and human relations [6–9]. This research has been undertaken across numerous disciplines, including public health, health economics, anthropology, sociology, human geography, and science and technology studies, and has taken a wide variety of forms, from stand-alone historical and ethnographic studies of technological innovation and intervention (e.g., [2, 10–12]), to numerous operational and evaluative studies of technical interventions (e.g., [13, 14]).

Yet to date there has been little systematic analysis of the context, scope, and extent of social research for specific global health technologies; to understand the kinds of social science questions that are commonly posed about global health technologies, the methods utilised to answer those questions, who undertakes the research, and to what purpose. Systematic mapping of the social research ecosystem in global health is important if we are to understand the full contribution made by this body of work, but also its limitations in terms of the voices and perspectives that are represented and the potentially important questions and topics omitted. In this paper, we provide a systematic evidence mapping of qualitative social research to answer these questions in one area: the development, deployment, and use of point-of-care tests (POCTs) in low-and middle-income country (LMIC) settings. Our aim is to prompt reflection on what qualitative social research is *for;* that is, what work we want it to do in global health.

POCT devices that do not rely on modern laboratory equipment or highly skilled staff, are portable and easy to use, and provide rapid turnover of results have the potential to transform healthcare in LMICs by dramatically improving access to and speed of diagnosis [15–18]. Strengthening diagnostic systems is widely viewed as essential not only to clinical management but also to tackling antimicrobial resistance, achieving universal health coverage, rising costs of new medicines, and emerging disease threats and epidemics [19–21]. In recent years, POCTs have become a near-ubiquitous feature of international disease control, elimination, and health systems strengthening programmes. The growing importance of POCTs to global health priorities is reflected in the release of the WHO Model List of Essential Diagnostics List (EDL) in 2018 [22, 23], which currently lists 32 POCTs as the basic diagnostic tests that should be made available for use in health facilities without laboratories [24, 25]. The COVID-19 pandemic heightened awareness of the importance of diagnostics and fuelled calls for global access to be accelerated and decentralised, for example through expanding the use of POCTs in community settings [26].

The ambition for expansion of POCTs in LMICs over the past three decades has inspired a substantial body of qualitative social research focused on these technologies. This body of research is yet to be assessed for whether it responds to the breadth of health priorities in countries, whether its funding and authorship reflect the full social and geographic spread of knowledge on this topic, and the scope of the research questions and objectives that underpin this research.

This systematic evidence mapping of qualitative social research focused on the development, deployment, and use of POCTs in LMICs, and was guided by two research questions:

- Research question 1: Where, by whom, and how has qualitative and social science research on POCTs in LMICs been produced?
- Research question 2*:* What is the scope and extent of existing qualitative and social science research on POCTs and what are the limitations?

We aim to provide a guide to the growing field of qualitative social research on diagnostics for social scientists, qualitative public health researchers, and global health actors by mapping its key contributions, strengths, weaknesses, and areas of density and sparsity. Systematic evidence mapping also has the potential to generate data on the social and economic conditions of knowledge production in global health research and provide insights to how those conditions might shape the scope of the evidence base. We argue that a reflexive approach to the role, status, and contribution of qualitative and social science research is crucial to identify the contributions it can make to the production of global health knowledge and understanding the roles technology can play in achieving global health goals.

## Methodology

### Search strategy

We conducted an exhaustive search of academic databases to identify studies related to ‘point-of-care testing’, ‘qualitative research’, and ‘low- and middle-income countries’. For each category, we identified multiple keywords taking inspiration from other scholars to develop searches for LMICs [27], POCTs [28], and qualitative research [29–31], and identified their index terms in bibliographic databases.

We searched the following databases: MEDLINE, Embase, Anthropology Plus, Web of Science, CINAHL, Scopus, Global Index Medicus, World Health Organization, Global Health (CABI), and ProQuest Dissertations and Theses Global database.

Guided by the University of Edinburgh Information Specialist (MD), we developed targeted search strategies for each database (see supplementary material (see Additional file 1). We used the PRESS checklist [32] to appraise our search strategies. We searched all databases for studies published from 1 January 2000 to 7 October 2022, as the field of POCTs is shifting so rapidly that we assumed evidence prior to this is less likely to be relevant to the contemporary context. In a subsequent search, we reviewed the references and citations of all papers meeting the inclusion criteria in the Web of Science to identify studies not initially picked up. We also manually searched books and journals less likely to be picked up in the search.

### Eligibility criteria

We included papers reporting on primary studies employing qualitative designs and exploring any POCT designed to detect health disorders in LMICs. We approached papers which reported on mixed-methods studies with caution. Although we did not carry out a specific search to identify mixed-methods studies, we included papers drawing from studies which were mixed-methods in design, but in which the qualitative component was epistemologically distinct from the quantitative component; i.e., demonstrating a non-positivist research epistemology, and in which the qualitative component was reported in a separate publication from the quantitative component of the study. We purposefully excluded studies which demonstrated a positivist epistemology since our aim was to focus on the contribution that interpretivist, constructivist, and critical ‘social’ epistemologies could offer as a balance to positivist epistemologies [6, 7].

We did not place any limits on language and did not exclude papers based on methodological limitations of the studies (see Additional file 2).

### Source selection

After carrying out the search, we removed duplicates from the set of identified papers. Using Covidence software, paper titles and abstracts were independently screened by two team members. Full texts of papers which passed this phase were retrieved and eligibility for inclusion independently assessed by two members of the team. Disagreements regarding inclusion or exclusion reason were reviewed by a third team member and then resolved through discussion.

### Data extraction and analysis

From each included study we extracted (a) publication data, i.e., author, year, title, funder, and author institutions, and (b) descriptive data, i.e., study context, methods, geographical locations, health conditions concerned, POCT type, biological sample, purpose of POCT, setting, stage(s) of the POCT life cycle studied and perspective(s) considered. Data were extracted from each paper by two team members and disagreements resolved through discussion.

Data were cleaned by another team member not involved in data extraction. We then used this data to generate descriptive analyses related to the categories of interest using Excel.

## Findings

### Included studies

**Fig. 1:**
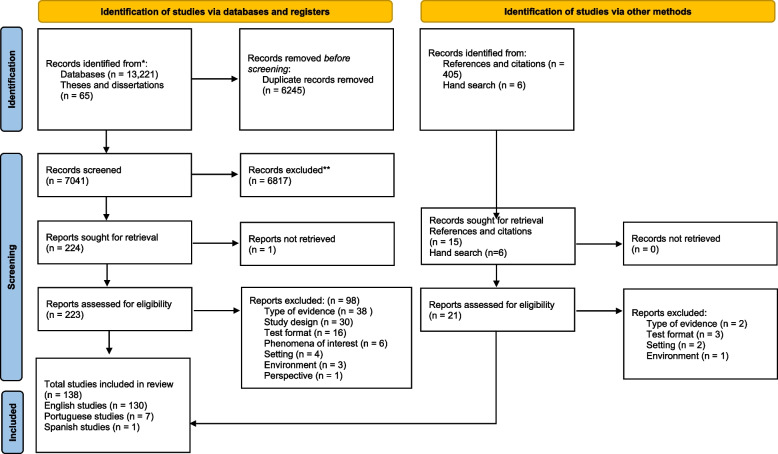
Prisma flow diagram

Figure 1 presents the PRISMA flow diagram of the paper search and selection. Through the database search, 13,221 papers were identified. After removing duplicates, 7041 papers remained for title and abstract screening, among which 224 met the inclusion criteria to move to full-text screening. Full text articles were retrieved for 223 articles, of which 125 met the inclusion criteria after full-text screening. Through the subsequent reference and citation search of the 125 included papers, we identified an additional 405 papers for screening. In the manual search, we identified six further papers and book chapters. After title and abstract screening of these 411 papers from the reference and citation and manual search, 21 were retrieved for full-text screening. Thirteen met the inclusion criteria. This resulted in a total of 138 articles included in the review. While most of the sources meeting our inclusion criteria were English-language, seven Portuguese and one Spanish paper met our criteria and were included in the analysis. Data from these papers were extracted and analysed by team members fluent in these languages.

### Spatial and temporal distribution of research

**Fig. 2:**
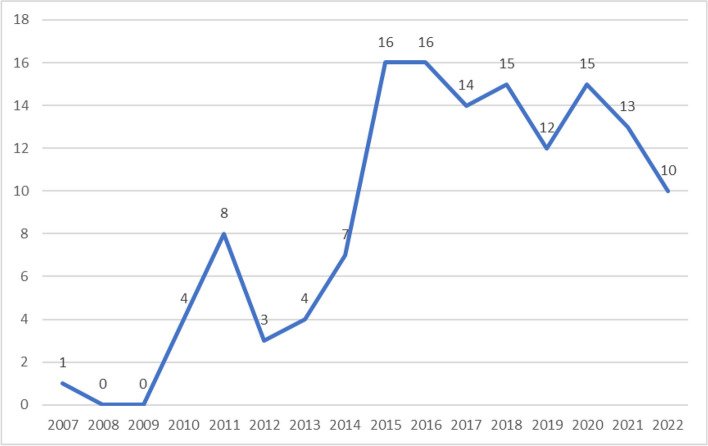
Chronology of included number of publications per year, January 2007-September 2022. 2022 data for January–September 2022 only

Figure 2 presents the number of papers meeting the inclusion criteria over time. No papers were included prior to 2007. Between four and eight papers were included for each year from 2010–14. For 2015 the number of included studies increased to 16, and between 12–16 studies were identified for each year from 2016 to 2021, suggesting a possible upward trend. Ten studies from the first nine months of 2022 were included.

**Fig. 3:**
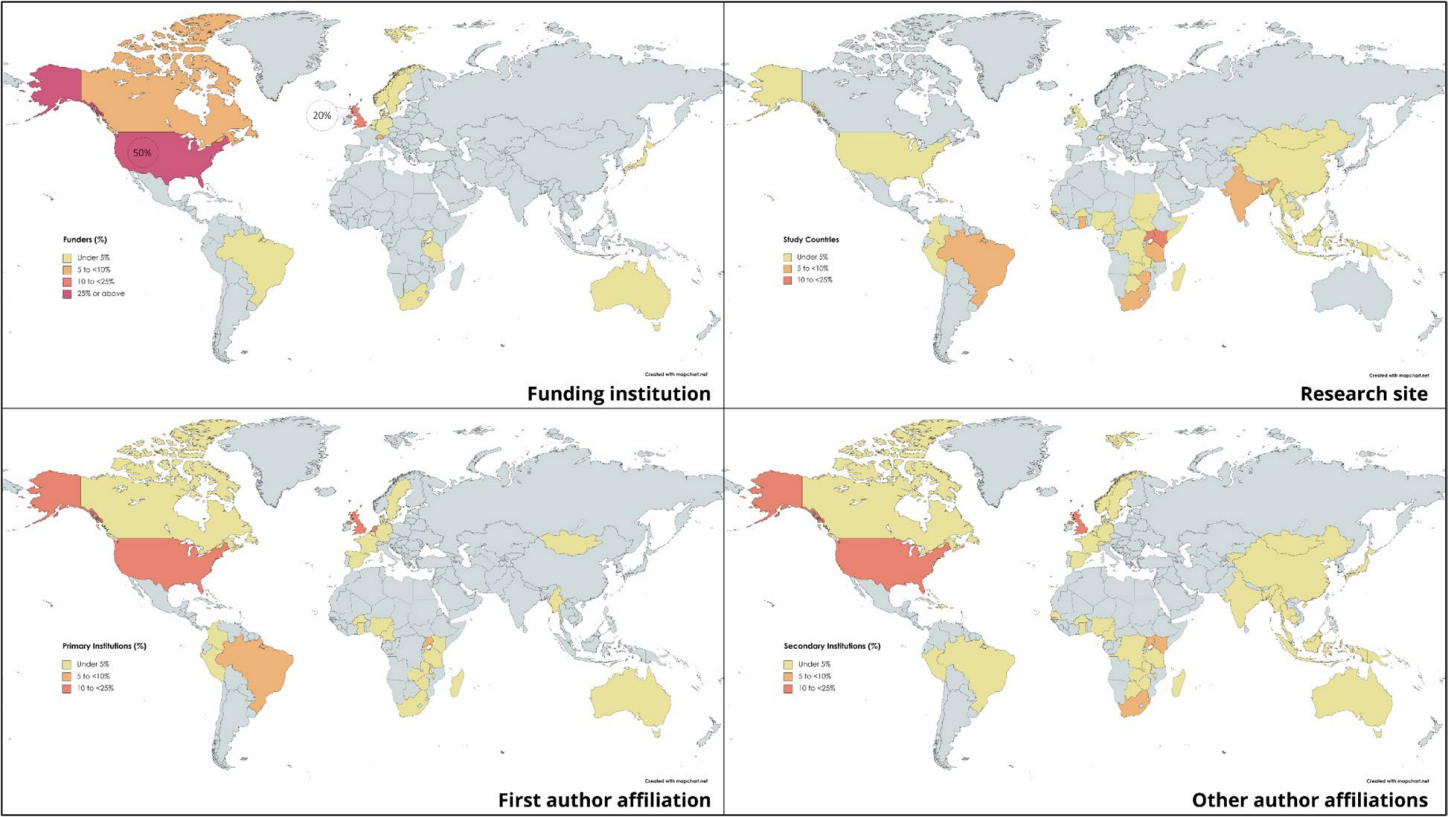
Study funding, site and authorship location

Figure 3 illustrates the geographic distribution of funding institutions, research sites and the locations of institutional affiliations for the first author and for all other authors of the papers.

The upper left-hand map of Fig. 3 presents the location of the funding institutions papers credited with providing financial support to the different studies. Across the 138 papers, 167 funding credits were reported, spanning 62 distinct organisations/institutions. Among the 167 reported funding institution credits, United Stated-based institutions were credited with providing financial support in 50% (*n* = 84) of cases, and United Kingdom-based institutions in 20% (*n* = 33) of cases. Twenty-five percent (*n* = 42) of the reported credits were of institutions located in other countries spanning high-income countries (HICs) (Australia, Belgium, Canada, Denmark, German, Japan, Netherlands, Norway, Sweden and Switzerland). Only four funding institutions based in LMICs were represented (Brazil, South Africa, Tanzania and Uganda), comprising 5% (*n* = 8) of the reported funding institution credits. The Bill and Melinda Gates Foundation was the most reported funding institution, credited in 20% (*n* = 27) of all papers. The United States National Institute of Health was the second most credited funding institution (*n* = 12; 9%) followed by the Wellcome Trust (*n* = 10; 7%).

The upper right-hand map in Fig. 3 represents the countries in which the studies reported by the papers were carried out (multiple possible). While 41 countries are represented on this map as study sites, the sites are highly concentrated, with nearly half (47%) of reported study sites located in the following five countries: Uganda (*n* = 22; 13%), Kenya (*n* = 18; 11%) South Africa (*n* = 15; 9%), Brazil (*n* = 13; 8%) and India (*n* = 10; 6%).

The two lower maps of Fig. 3 represent the institutional affiliation of paper authors. The lower left-hand map presents the location of the first author affiliation. Across the papers, 148 first author affiliations were mentioned, with first authors on 10 papers providing dual-affiliations. The majority of first author affiliations were located in HICs (*n* = 94; 64%), most commonly in the United Kingdom (*n* = 27; 18%), the United States (*n* = 24; 16%) and the Netherlands (*n* = 16; 11%). The University of Maastricht, located in the Netherlands, was the single most represented institution. Three scholars affiliated with this university were first author on 14 papers (9%), and one of these authors was first author on 10 of these papers, and senior author on the other four. First author affiliations located in LMIC-based institutions were mentioned in 35% (*n* = 54) of studies, most commonly in Brazil (*n* = 11; 7%) and Uganda (*n* = 7; 5%).

The lower right-hand map illustrates the location of affiliated institutions for all other authors besides the first author. Across the papers, there were 438 mentions of affiliated institutions for all non-first authors. Compared to first author affiliations, these were more evenly divided between LMICs (*n* = 243; 53%) and HICs (*n* = 204; 47%).

### Research context and approach

**Fig. 4:**
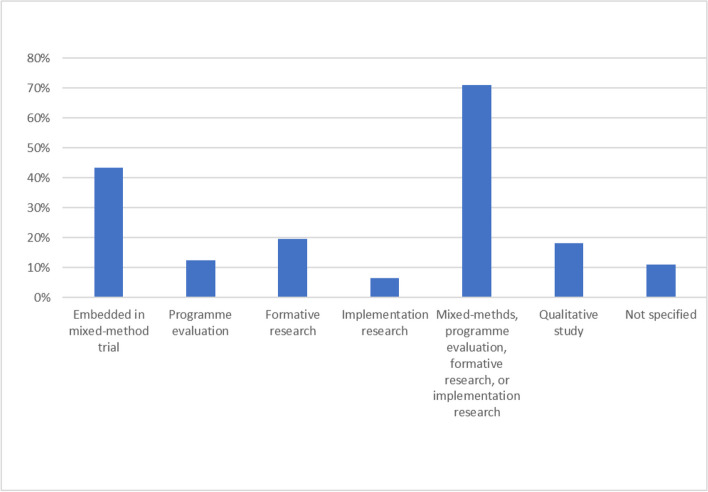
Reported study context

Figure 4 presents the wider research context reported. The majority (*n* = 98; 71%) of papers reported qualitative research to be embedded within implementation projects or trials. Twenty-five (18%) studies were reported as solely qualitative and not explicitly attached to a trial or intervention.

Interviews were the most reported research method, with 88% (*n* = 122) of papers reporting interviews as among the methods employed in studies. The second most common method was focus group discussions (*n* = 60; 43%). Fourteen percent (*n* = 20) of papers mentioned drawing from ethnographic methods, including in-depth ethnographic fieldwork (*n* = 12, 9%), focused ethnographic methods (*n* = 5, 4%), or using participant observation as a method (*n* = 3, 2%) (see Additional file 3). Of these, 10 (7%) were reported as a study embedded within an intervention or trial, eight (6%) were reported as part of an independent qualitative study, and in two (1%), the context was unspecified.

### Scope of research (object of study)

Table 1 presents the health conditions and tests which were reported as the object of studies. HIV (*n* = 68; 49%) and malaria (*n* = 50; 36%) were the most commonly reported health conditions, with over 80% (*n* = 114) of papers reporting on one or both health conditions. Over half of studies (*n* = 40/68; 59%) examining POCTs for diagnosing HIV examined HIV rapid diagnostic tests (HIV RDTs), which were represented in 30% of all papers, while nearly all (*n* = 47/50; 94%) malaria-focused papers examined the use of malaria rapid diagnostic tests (mRDTs), represented in 35% of all papers overall. Papers reporting on malaria testing peaked in 2016, with 11 papers reporting on malaria published that year, decreasing to one and two papers in 2020 and 2021, respectively (no such paper published in 2022 was included up to the date of search). In contrast, papers reporting on HIV peaked in 2020 at 12, and remained high, with seven HIV-reporting papers in 2021–22, the highest reported condition over these years (see Additional files 4 and 5).

**Table 1:** Reported health conditions and tests

Condition	# of studies	% of Studies	Test	# of studies	% (of studies related to the condition)	Studies
HIV	68	49%	HIV RDT	40	59%	5,7,9,11,16,17,18,19,26,27,30,31,32,34, 35,36,38,40,42,43,46,47,57,66,68,69,85, 89,90,95,101,102,112,114,115,119,120, 132,135,136
			HIVST	14	21%	8,11,17,23,55,56,57,59,66,69,90,96,103, 127
			EID POC	10	15%	63,74,81,88,108,121,129,130, 133, 134
			CD4 POC	8	12%	25,35,36,54,67,83,109,110
			HIV/Syphilis dual RDT	1	1%	75
			TFV UT	1	1%	122
			Not specified	1	1%	33
Malaria	50	36%	mRDT	47	94%	1,2,3,6,10,12,13,14,15,20,21,22,24,28,32, 41,48,49,50,51,52,53,58,60,73,79,82,84, 91,92,93,94,97,98,99,104,107,111,113, 116,117,123,124,125,131,135,138
			G6PD RDT	1	2%	71
			Not specified	2	4%	30,33
Syphilis	14	10%	Syphilis RDT	9	64%	5,11,16,27,31,69,95,100,135
			HIV/Syphilis dual RDT	1	7%	75
			VDRL Card test	1	7%	32
			DPP-POCT	1	7%	76
			Not specified	1	7%	7
TB	11	8%	TB Xpert MTB/RIF	8	73%	31,35,36,37,38,80,87,106
			LAM POC	1	9%	86
			MTBDRplus	1	9%	87
			Not specified	2	18%	29,33
Dengue	6	4%	Dengue RDT	3	50%	12,98,137
			Not specified	3	50%	30,32,33
Bacterial infection	5	4%	CRP POCTs	4	80%	44,45,65,126
			Not specified	1	20%	39
Diabetes	4	3%	Glucometer	2	50%	30,32
			Blood Glucose (finger prick)	1	25%	31
			Not specified	1	25%	33
Hepatitis	3	2%	HCV Self-test	1	33%	77
			HbsAG Card (hepatitis)	1	33%	32
			Not specified	1	33%	16
Anaemia	3	2%	Anaemia RDT	1	33%	135
			HCS	1	33%	97
			Not specified	1	33%	7
COVID-19	3	2%	COVID-19 RDT	3	100%	61,64,126
Typhoid	2	1%	Not specified	2	100%	30,32
Zika	2	1%	Zika RDT	2	100%	62,64,
Ebola	1	1%	Ebola RDT	1	100%	64
HAT	1	1%	HAT RDT	1	100%	70
Leishmaniasis	1	1%	leishmaniasis RDT	1	100%	118
Meningitis	1	1%	CrAG LFA	1	100%	72
Urinary infections	1	1%	Not specified	1	100%	39
Yaws	1	1%	DDP-POCT	1	100%	76
Not specified	7	5%	Not specified	7	100%	4,30,31,32,33,78,105

Syphilis testing was represented in 10% (*n* = 14) of papers, while 8% (*n* = 11) of papers reported on studies examining tuberculosis (TB) testing.

Among reported health conditions, many papers focused on testing in the context of antenatal care, including: seven out of 68 papers on HIV testing; eight out of the 50 papers on malaria testing; and five out of 14 papers on syphilis testing.

The most common test format considered were RDTs, reported in 71% (*n* = 98) of papers, followed by molecular tests, reported on in 10% (*n* = 14) of papers (see Additional file 6).

**Fig. 5:**
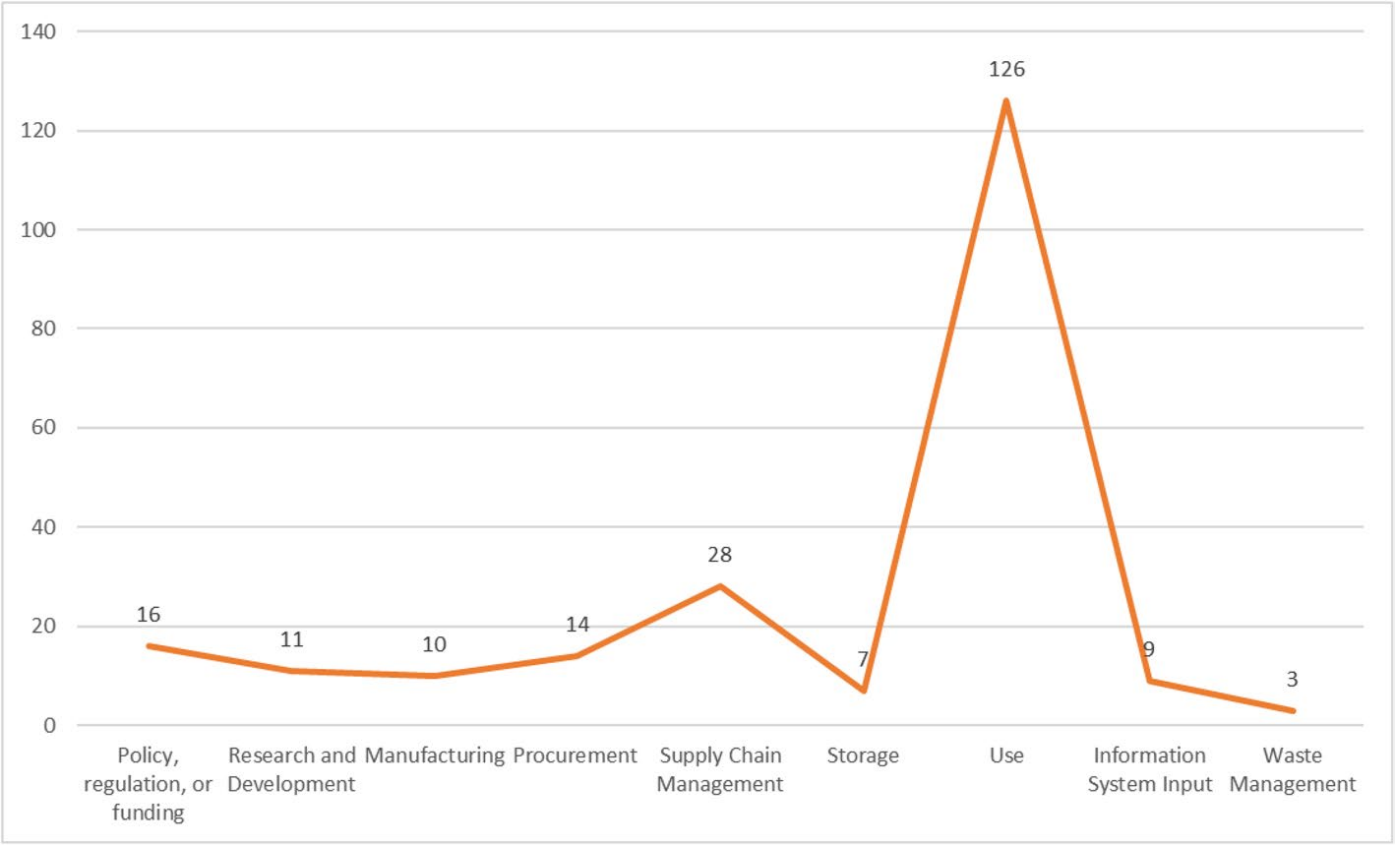
POCT life cycle moment represented

Figure 5 presents the moments across the POCT life cycle represented in the included papers.^1^ Similar numbers of papers reported on policy, regulation, or funding (*n* = 16; 12%), manufacturing (*n* = 10; 7%) and procurement (*n* = 14; 10%). Twenty-eight (20%) papers report on supply chain management and seven (5%) on storage. Almost all (*n* = 126; 91%) papers concerned the moment of POCT use, e.g., taking and testing the sample, interpreting the results, and post-result decision-making and action. Nine (7%) studies considered input of information generated with POCTs into information systems, while only three considered waste management and disposal.

Primary health care facilities were the most represented settings, reported on in 60% (*n* = 83) of papers. Hospitals (*n* = 41; 30%) were the second most common setting, followed by community settings (*n* = 37; 27%), and domestic spaces (*n* = 20; 15%) (see Additional file 7). Among the papers which specified the setting as either a public/state setting or a private/commercial setting (*n* = 99, 72%), 78% (*n* = 77) reported on studies carrying out research in publicly funded health settings only, 9% (*n* = 9) in private/commercial settings only, while 13% (*n* = 13) reported on studies carried out in both public and private/commercial health settings (see Additional file 8).

While the body of papers took into account perspectives of actors across the POCT life cycle, the most densely represented perspectives were those of the formal health workforce, with 75% of papers taking these perspectives into account. The second most represented perspective was that of patients (*n* = 41; 30%), while 15% (*n* = 21) considered the perspective of patients’ family members. Fewer papers considered perspectives of policymakers, regulators, developers or designers, and specific end-user groups considered “at risk” or “vulnerable” (see Additional file 9).

## Discussion

The findings of this mapping indicate that qualitative social research related to POCTs for LMICs is highly concentrated and displays a striking lack of diversity and breadth, both in terms of how, where and by whom it has been produced, and the scope of research. Qualitative social research exploring POCTs for LMICs is often deployed to support quantitative scientific studies rather than contribute to the production of knowledge in the social sciences. While we did not search for mixed-methods studies, nearly half of included papers were reported as companions to a quantitative study—over double those reported as solely qualitative research. This is perhaps unsurprising since global health research is historically rooted in the traditions of clinical research, which privileges quantitative research and is underpinned by positivist epistemologies. Though important, positivist epistemologies are limited in their ability to produce knowledge related to the human condition, perceptions and experiences, or to reflexively interrogate underpinning value assumptions and cultural frameworks. Qualitative social research, which is underpinned by interpretive epistemologies, is especially well-equipped to contribute to these areas [6, 7, 33]. It is therefore important to consider how the appendage of qualitative studies to quantitative studies may shape, and potentially constrain, the knowledge they generate [33–36].

The context of the reported studies—including the funding sources, where research is done, by whom, and where research is published—is important for understanding the global health research ecosystem within which the purpose and scope of research is formulated, [37] A majority of papers reported on studies carried out within the context of a trial or intervention, suggesting that qualitative research has been brought in primarily to answer questions about implementation-related variables, e.g., feasibility and acceptability, and potentially omitting critical questions that social scientists might pose, such as those pertaining to the broader structural conditions in which POCTs are prioritised, funded, developed and deployed, or the ways in which POCTs reconfigure power relationships in medicine (see e.g., [38–42]). Ethnographic methods are especially well-suited to unearthing these social and political dimensions of global health technologies [9, 43–45] and have been shown to be essential to better understand the lived experience and everyday workings of health systems and technologies in real-world settings (e.g., [2, 10–12]). However, these methods were employed in only a small proportion of studies reported in these papers.

Our analysis reveals stark geographic disparities in the research ecosystem in terms of funding, author affiliation, and the location of study sites. Study sites were highly concentrated, with nearly half of reported study sites located in only five LMICs. As most studies were a companion to an intervention or trial, the narrowness of study locations is likely driven by funding priorities, and existing partnerships of HIC-based institutions. Not only is funding concentrated in a few HICs, with fully half of all funding credits to institutions based in the United States alone, it is also concentrated among a narrow group of institutions. The Bill and Melinda Gates Foundation emerged as the dominant funding institution, credited with providing funding for one in every five papers. Arguably, no other entity has been more instrumental in setting the terms in the transition from international to global health and driving technological solutions to global health [46–49]. Yet there is potential for important research topics and questions to be occluded when research funding on a technology is controlled by actors who are also its proponents and funders [50, 51].

The geographical distribution of author institutions is also revealing. In recent years, numerous calls have been made to decolonise global health research and address the “epistemic injustice” of moral wrongs which occur in relation to knowledge production, use, and circulation, in global health [52–54]. One pathway towards epistemic justice involves attention to inclusive authorship [55–57]. First authorship is often considered a proxy for leadership of a paper [57], and our mapping highlights the striking density of first authorship affiliation in HIC-based institutions, with approximately two-thirds of first author affiliations based in HICs. For all other author institutions, the margin was much narrower between LMIC- and HIC-based institutions, with only slightly more author affiliations located in HICs. The close parity between HICs and LMICs in terms of non-first authorship might be seen as progress in authorial equity, although several studies have highlighted the risk that authors from LMICs get ‘stuck-in-the middle’ of the author list, or that their inclusion becomes symbolic ‘guest authorship’ instead of a genuine opportunity to make a conceptual and analytical contribution, as scientific authorship usually entails [52, 54, 58–61].

Our findings likely reflect inequities in both the geographic distribution of research funding and additional institutional infrastructures, resources, and support available to scholars in HICs when compared to scholars in LMICs. The authorship of this paper is a case in point and indicates the difficulty of addressing these inequities within current structures of employment and funding, even when researchers are aware of current debates around decolonising global health [62–64]. Broadening authorship can ensure that the questions posed and interpretation of results are relevant to global health practice in LMICs and enhance the accessibility of results to LMIC audiences.

At a structural level, these geographic disparities might also be viewed as a symptom of a persistent colonial legacy within global health [65]. Our findings therefore illustrate the continued importance of addressing equity in research expertise on global health technologies as part of broader agendas to decolonise global health. Building research capacity in LMICs to drive global health research is crucial to advancing epistemic justice and addressing global health challenges [66–69], and investing in LMIC capacity for interpretive and critical social science research in particular, with its commitment to the philosophical and social justice principles fundamental to decolonisation [70], may open promising pathways towards expanding epistemic justice.

If the conditions of knowledge production in this field are heavily circumscribed, our findings show that the scope of this body of research is equally narrow. The research we mapped is overwhelmingly skewed toward two health conditions: HIV and malaria. While this may be explained by these being some of the earliest introduced tests, this likely also reflects the extent to which qualitative social research has followed the funding priorities of HIC-based global health institutions, for which malaria and HIV have been priorities for several decades. It is notable that very few studies focused on non-communicable diseases, despite these increasingly contributing to disease burdens in LMICs [71, 72], and many tests considered ‘essential’ by the WHO’s Essential Diagnostic List [24], such as those for TB, diabetes and syphilis in pregnancy, and hepatitis B infection during pregnancy, are under-represented or not represented at all in the included studies. Neglected tropical diseases (NTDs) were also under-represented, with only three papers reporting of use of POCTs for NTDs, with human African trypanosomiasis, leishmaniasis, and yaws considered in one paper each. This represents an important gap, with expanding access to POCTs for diagnosing NTDs a core component of the global road map to prevent, control, eliminate, or eradicate 20 NTDs by 2030 [73].

Papers overwhelmingly reported on the moment in which POCTs were used, leaving knowledge related to other lifecycle moments marginalised, such as how tests are developed, manufactured and regulated, and supplied, and issues around waste management in contexts in which waste management is known to be especially challenging [74]. The papers also reported primarily on POCT use in public health settings; a particularly glaring blind spot given that private health services are becoming dominant in health service delivery throughout LMICs [75]. It is critical to understand how the value of POCTs are transformed in health marketplaces and what this means for how they are used and how they affect follow-up action.

Although the number of qualitative research publications exploring POCTs in LMICs has increased year-on-year since 2007, fewer than 20 studies were published each year, suggesting that qualitative social research remains an underused resource in this area. However, the findings of this mapping also indicate striking densities of evidence and substantial blind spots in qualitative social research on global health technologies such as POCTs. In addition to increasing the quantity of qualitative research in this area, and LMIC capacity to drive this research, we therefore argue that it is equally important to increase the breadth of research, including across test type, geographies, settings and perspectives, in order to fully understand the social relations and dynamics involved in their development and deployment, and the implications for global health access and equity.

Indeed, we suggest that a potential link might be drawn between our finding that the conditions of knowledge production in qualitative social research on POCTs in LMICs are narrow, and the finding that the scope of that research is equally limited in breadth. The conditions of knowledge production created by the research ecosystem shape all phases of research, from conceptualisation, to carrying out the research, analysis and interpretation, to who ultimately has access to it. Reflecting on and addressing inequities, imbalances, and omissions in the conditions of knowledge production is therefore crucial for purposes of epistemic justice and scientific integrity.

## Limitations

An important limitation of our study was our inability to adequately capture research generated in an intra- and post-COVID-19 context. COVID-19 was monumental in generating momentum for and normalising the decentralised use of POCTs, yet our search identified surprisingly few papers reporting on studies related to COVID-19 testing in LMICs. While COVID-19-related studies tended to be fast-tracked and prioritised, it seems that few entirely qualitative studies were published through these routes. We assume that this is at least in part due to the slower nature of producing qualitative evidence [76, 77].

Another limitation of the study is that we did not search for studies published before 2000. It is possible that we missed some early work, as some POCTs were in circulation prior to this. However, we are confident that few studies would have been missed, given that the earliest study identified in our search was published in 2007.

A further limitation of this study remains that its authorship is based primarily in HIC contexts. Indeed, authors from HICs have posed the questions, determined the methods, and interpreted the results even as we critique the predominance of HIC institution density in the generation of research. It also reflects the interpretations and conclusions of social scientists, and the desire to see more research that draws from social science epistemologies—work that is currently more often carried out in HICs than LMICs.

## Conclusion

This mapping exercise examined the conditions of qualitative knowledge production and global health research ecosystem related to one quintessential area of technology-driven global health practice—POCTs for use in LMICs. While numerous qualitative and social science researchers have responded to the call to generate knowledge related to the development, deployment, and use of diagnostics in LMICs, the overall body of research that has emerged from these efforts is highly concentrated in scope and overwhelmingly focuses on testing in the context of a narrow number of donor-supported vertical disease-control programmes. One potential factor in the limited breadth and the depth of research in this area, we argue, has been the predominant contextualisation of qualitative research within quantitative trial and intervention studies, driven largely by HICs through funding streams and expertise.

To optimise the potential contributions of qualitative research in global health, we suggest that successful research ecosystems would enable researchers to broaden their inquiries, in terms of their scope, underpinning epistemologies, and how studies are carried out. For POCTs in LMICs, this includes consideration of a wider range of health conditions, tests, settings and perspectives, particularly outside of trial contexts, driven by priorities and questions posed by LMIC actors, as well as a more expansive use of qualitative methods. This would require increased investment in social science capacity in LMICs, and, given the current structures of the global health political economy, a recognition by donors of the importance of building up such capacity and moving beyond a conceptualisation of qualitative social research as an adjunct to vertical programmes. A rich, diverse, and equitable research ecosystem is essential to move beyond siloed perspectives, foster critical perspectives on global health technologies and health systems strengthening, and formulate new understandings of and solutions to global inequities in access to health care.

### Supplementary Information

**Additional file 1.** Search Strategies.

**Additional file 2.** Selection criteria.

**Additional file 3.** Reported research methods.

**Additional file 4.** Chronology of reported health conditions.

**Additional file 5.** Chronology of HIV and malaria-reporting papers.

**Additional file 6.** Reported test format.

**Additional file 7.** Study setting.

**Additional file 8.** Reported settings as public or private.

**Additional file 9.** Reported perspectives.

**Additional file 10.** Summary of included papers.

## Data Availability

The datasets generated and analysed during the current study will be made available on the Edinburgh DataShare digital repository. The protocol for this study is registered with Prospero, ID number: CRD42022366518. It can be accessed at: https://www.crd.york.ac.uk/prospero/display_record.php?ID=CRD42022366518. See also Additional file 10 for a summary of the papers included in this evidence mapping.

## Footnotes

1 While this figure suggests a linearity of the POCT life cycle, we recognise the life cycle as not linear, but iterative, with particular moments informing other moments across the cycle (see also Engel, 2020).
