# Supplementary material for: Is qualitative social research in global health fulfilling its potential?: a systematic evidence mapping of research on point-of-care testing in low- and middle-income contexts
Source: BMC Health Serv Res. 2024 Feb 7;24:172. doi: 10.1186/s12913-024-10645-5 (PMC10848363; doi:10.1186/s12913-024-10645-5)
Supplement: Supplementary file 1 — Additional file 1. Search Strategies. [file 12913_2024_10645_MOESM1_ESM.pdf]

Additional File 1: Search Strategies

For our search, we developed targeted search strategies for each data base using index terms for 'point-of-care testing', 'qualitative research', and 'low- and middle-income countries'.

We drew inspiration from the following studies to inform our search strategy development:

For qualitative search terms:

Walters LA, Wilczynski NL, Haynes RB. (2006) Developing Optimal Search Strategies for Retrieving Clinically Relevant Qualitative Studies in EMBASE. Qualitative Health Research;16(1):162-168. doi:10.1177/1049732305284027.

Wilczynski NL, Marks S, Haynes RB. (2007) Search Strategies for Identifying Qualitative Studies in CINAHL. Qualitative Health Research; 17(5):705-710. doi:10.1177/1049732306294515

Wong SS, Wilczynski NL, Haynes RB; Hedges Team. (2004) Developing optimal search strategies for detecting clinically relevant qualitative studies in MEDLINE. Studies in Health Technology Informatics;107(Pt 1):311-6. PMID: 15360825.

For 'point-of-care testing' search terms:

Van Hecke, Oliver, et al. (2020). In-vitro diagnostic point-of-care tests in paediatric ambulatory care systematic review and meta-analysis. PLoS One 15(7):e0235605.

For LMIC search terms:

Papola D, Purgato M, Gastaldon C, Bovo C, van Ommeren M, Barbui C, Tol WA. (2020) Psychological and social interventions for the prevention of mental disorders in people living in low- and middle-income countries affected by humanitarian crises. Cochrane Database of Systematic Reviews 2020, Issue 9. Art. No.: CD012417. DOI: 10.1002/14651858.CD012417.pub2.

This review used the World Bank classification 2009 for their search strategy. We updated this by removing the following countries which are considered high income countries according to the World Bank fiscal year 2023 classification (https://datahelpdesk.worldbank.org/knowledgebase/articles/906519-world-bank-country-and-lending-groups): Chile, Croatia Czechoslovakia OR "Czech Republic", Cyprus, Estonia, Greece, Guam, Hungary, "Isle of Man", Latvia, Lithuania, Malta, Poland, Portugal, Romania or Rumania or Roumania, Seychelles, Slovakia OR "Slovak Republic", Slovenia.

Medline

1	Qualitative Research/
2	(interview* OR experience OR qualitative).mp.
3	(ethnograph* OR "ethnographic research").mp.
4	(phenomenol* OR "phenomenological research").mp
5	local concept*.mp.
6	Grounded Theory/
7	Focus Groups/
8	"purposive sampl*".mp.
9	("content analys*" OR "thematic analys*").mp.
10	"Observational method*".mp.
11	"case stud*".mp.

12	"action research".mp.
13	("conversation analys*" OR "discourse analys*").mp.
14	reveal.ti,ab,kw
15	1 or 2 or 3 or 4 or 5 or 6 or 7 or 8 or 9 or 10 or 11 or 12 or 13 or 14
16	(Africa or Asia or Caribbean or "West Indies" or "South America" or "Latin America" or "Central America").ti,ab,kw.
17	(Afghanistan OR Albania OR Algeria OR Angola OR Antigua OR Barbuda OR Argentina OR Armenia OR Armenian OR Aruba OR Azerbaijan OR Bahrain OR Bangladesh OR Barbados OR Benin OR Byelarus OR Byelorussian OR Belarus OR Belorussian OR Belorussia OR Belize OR Bhutan OR Bolivia OR Bosnia OR Herzegovina OR Hercegovina OR Botswana OR Brasil OR Brazil OR Bulgaria OR "Burkina Faso" OR "Burkina Fasso" OR "Upper Volta" OR Burundi OR Urundi OR Cambodia OR "Khmer Republic" OR Kampuchea OR Cameroon OR Cameroons OR Cameron OR Camerons OR "Cape Verde" OR "Central African Republic" OR Chad OR China OR Colombia OR Comoros OR "Comoro Islands" OR Comores OR Mayotte OR Congo OR Zaire OR "Costa Rica" OR "Cote d'Ivoire" OR "Ivory Coast" OR Cuba).ti,ab,kw.
18	(Djibouti or "French Somaliland" or Dominica or "Dominican Republic" or "East Timor" or "East Timur" or "Timor Leste" or Ecuador or Egypt or "United Arab Republic" or "El Salvador" or Eritrea or Ethiopia or Fiji or Gabon or "Gabonese Republic" or Gambia or Gaza or Georgia or Georgian or Ghana or "Gold Coast" or Grenada or Guatemala or Guinea or Guiana or Guyana or Haiti or Honduras or Hungary or India or Maldives or Indonesia or Iran or Iraq or "Isle of Man" or Jamaica or Jordan or Kazakhstan or Kazakh or Kenya or Kiribati or Korea or Kosovo or Kyrgyzstan or Kirghizia or "Kyrgyz Republic" or Kirghiz or Kirgizstan or "Lao PDR" or Laos or Lebanon or Lesotho or Basutoland or Liberia or Libya).ti,ab,kw.
19	(Macedonia or Madagascar or "Malagasy Republic" or Malaysia or Malaya or Malay or Sabah or Sarawak or Malawi or Nyasaland or Mali or "Marshall Islands" or Mauritania or Mauritius or "Agalega Islands" or Mexico or Micronesia or "Middle East" or Moldova or Moldovia or Moldovian or Mongolia or Montenegro or Morocco or Ifni or Mozambique or Myanmar or Myanma or Burma or Namibia or Nepal or "Netherlands Antilles" or "New Caledonia" or Nicaragua or Niger or Nigeria or "Northern Mariana Islands" or Oman or Muscat or Pakistan or Palau or Palestine or Panama or Paraguay or Peru or Philippines or Philipines or Phillipines or Phillippines or "Puerto Rico").ti,ab,kw.
20	(Russia or Russian or Rwanda or Ruanda or "Saint Kitts" or "St Kitts" or Nevis or "Saint Lucia" or "St Lucia" or "Saint Vincent" or "St Vincent" or Grenadines or Samoa or "Samoan Islands" or "Navigator Island" or "Navigator Islands" or "Sao Tome" or "Saudi Arabia" or Senegal or Serbia or Montenegro or "Sierra Leone" or "Sri Lanka" or Ceylon or "Solomon Islands" or Somalia or Sudan or Suriname or Surinam or Swaziland or Syria or Tajikistan or Tadzhikistan or Tadjikistan or Tadzhik or Tanzania or Thailand or Togo or "Togolese Republic" or Tonga or Trinidad or Tobago or Tunisia or Turkey or Turkmenistan or Turkmen or Uganda or Ukraine or Uruguay or USSR or "Soviet Union" or "Union of Soviet Socialist Republics" or Uzbekistan or Uzbek or Vanuatu or "New Hebrides" or Venezuela or Vietnam or "Viet Nam" or "West Bank" or Yemen or Yugoslavia or Zambia or Zimbabwe or Rhodesia).ti,ab,kw.
21	((developing or "less* developed" or "under developed" or underdeveloped or "middle income" or "low* income" or underserved or "under served" or deprived or poor*) adj1 (countr* or nation* or population* or world)).ti,ab,kw.

22	((developing or "less* developed" or "under developed" or underdeveloped or "middle income" or "low* income") adj1 (economy or economies)).ti,ab,kw.
23	(low* ADJ1 (GDP or GNP or "gross domestic" OR "gross national")).ti,ab,kw.
24	(low ADJ3 middle ADJ3 countr*).ti,ab,kw.
25	(LMIC OR LMICs OR "third world" OR "LAMI country" OR "LAMI countries").ti,ab,kw.
26	("transitional country" OR "transitional countries").ti,ab,kw.
27	16 or 17 or 18 or 19 or 20 or 21 or 22 or 23 or 24 or 25 or 26
28	Point-of-Care Systems/
29	Point-of-Care Testing/
30	(("point of care" or POC) and (test* or diagnos*)).ti,ab,kw.
31	poct.ti,ab,kw.
32	((rapid or bedside or bed-side or "near patient") and (test* or diagnos*)).ti,ab,kw.
33	"outbreak setting".ti,ab,kw.
34	28 or 29 or 30 or 31 or 32
35	15 and 27 and 34
36	limit 35 to yr="2000 -Current"

Embase

1	Qualitative research/
2	(interview* or experience or qualitative).ti,ab,kw.
3	Interview/
4	Ethnography/
5	Phenomenology/
6	local concept*.ti,ab,kw.
7	Grounded Theory/
8	focus group*.ti,ab,kw.
9	Purposive sample/
10	Content analysis/
11	Thematic analysis/
12	Observational method/
13	Case study/
14	case stud*.ti,ab,kw.
15	action research.ti,ab,kw.
16	conversation analys*.ti,ab,kw.
17	Discourse analysis/
18	reveal.ti,ab.
19	1 or 2 or 3 or 4 or 5 or 6 or 7 or 8 or 9 or 10 or 11 or 12 or 13 or 14 or 15 or 16 or 17 or 18
20	(Africa or Asia or Caribbean or "West Indies" or "South America" or "Latin America" or "Central America").ti,ab,kw.

21	(Afghanistan or Albania or Algeria or Angola or Antigua or Barbuda or Argentina or Armenia or Armenian or Aruba or Azerbaijan or Bahrain or Bangladesh or Barbados or Benin or Byelarus or Byelorussian or Belarus or Belorussian or Belorussia or Belize or Bhutan or Bolivia or Bosnia or Herzegovina or Hercegovina or Botswana or Brasil or Brazil or Bulgaria or "Burkina Faso" or "Burkina Fasso" or "Upper Volta" or Burundi or Urundi or Cambodia or "Khmer Republic" or Kampuchea or Cameroon or Cameroons or Cameron or Camerons or "Cape Verde" or "Central African Republic" or Chad or China or Colombia or Comoros or "Comoro Islands" or Comores or Mayotte or Congo or Zaire or "Costa Rica" or "Cote d'Ivoire" or "Ivory Coast" or Cuba).ti,ab,kw.
22	(Djibouti or "French Somaliland" or Dominica or "Dominican Republic" or "East Timor" or "East Timur" or "Timor Leste" or Ecuador or Egypt or "United Arab Republic" or "El Salvador" or Eritrea or Ethiopia or Fiji or Gabon or "Gabonese Republic" or Gambia or Gaza or Georgia or Georgian or Ghana or "Gold Coast" or Grenada or Guatemala or Guinea or Guiana or Guyana or Haiti or Honduras or Hungary or India or Maldives or Indonesia or Iran or Iraq or "Isle of Man" or Jamaica or Jordan or Kazakhstan or Kazakh or Kenya or Kiribati or Korea or Kosovo or Kyrgyzstan or Kirghizia or "Kyrgyz Republic" or Kirghiz or Kirgizstan or "Lao PDR" or Laos or Lebanon or Lesotho or Basutoland or Liberia or Libya).ti,ab,kw.
23	(Macedonia or Madagascar or "Malagasy Republic" or Malaysia or Malaya or Malay or Sabah or Sarawak or Malawi or Nyasaland or Mali or "Marshall Islands" or Mauritania or Mauritius or "Agalega Islands" or Mexico or Micronesia or "Middle East" or Moldova or Moldovia or Moldovian or Mongolia or Montenegro or Morocco or Ifni or Mozambique or Myanmar or Myanma or Burma or Namibia or Nepal or "Netherlands Antilles" or "New Caledonia" or Nicaragua or Niger or Nigeria or "Northern Mariana Islands" or Oman or Muscat or Pakistan or Palau or Palestine or Panama or Paraguay or Peru or Philippines or Philipines or Phillipines or Phillippines or "Puerto Rico").ti,ab,kw.
24	(Russia or Russian or Rwanda or Ruanda or "Saint Kitts" or "St Kitts" or Nevis or "Saint Lucia" or "St Lucia" or "Saint Vincent" or "St Vincent" or Grenadines or Samoa or "Samoan Islands" or "Navigator Island" or "Navigator Islands" or "Sao Tome" or "Saudi Arabia" or Senegal or Serbia or Montenegro or "Sierra Leone" or "Sri Lanka" or Ceylon or "Solomon Islands" or Somalia or Sudan or Suriname or Surinam or Swaziland or Syria or Tajikistan or Tadzhikistan or Tadjikistan or Tadzhik or Tanzania or Thailand or Togo or "Togolese Republic" or Tonga or Trinidad or Tobago or Tunisia or Turkey or Turkmenistan or Turkmen or Uganda or Ukraine or Uruguay or USSR or "Soviet Union" or "Union of Soviet Socialist Republics" or Uzbekistan or Uzbek or Vanuatu or "New Hebrides" or Venezuela or Vietnam or "Viet Nam" or "West Bank" or Yemen or Yugoslavia or Zambia or Zimbabwe or Rhodesia).ti,ab,kw.
25	((developing or "less* developed" or "under developed" or underdeveloped or "middle income" or "low* income" or underserved or "under served" or deprived or poor*) adj1 (countr* or nation* or population* or world)).ti,ab,kw.
26	((developing or "less* developed" or "under developed" or underdeveloped or "middle income" or "low* income") adj1 (economy or economies)).ti,ab,kw.
27	(low* adj1 (GDP or GNP or "gross domestic" or "gross national")).ti,ab,kw.
28	(low adj3 middle adj3 countr*).ti,ab,kw.
29	(LMIC or LMICs or "third world" or "LAMI country" or "LAMI countries").ti,ab,kw.
30	("transitional country" or "transitional countries").ti,ab,kw.

31	20 or 21 or 22 or 23 or 24 or 25 or 26 or 27 or 28 or 29 or 30
32	Point of care system/
33	Point of care testing/
34	(("point of care" or POC) and (test* or diagnos*)).ti,ab,kw.
35	poct.ti,ab,kw.
36	((rapid or bedside or bed-side or "near patient") and (test* or diagnos*)).ti,ab,kw.
37	rdt.ti,ab,kw.
38	outbreak setting.ti,ab,kw.
39	32 or 33 or 34 or 35 or 36 or 38
40	19 and 31 and 39
41	limit 40 to yr="2000 -Current"

Global Health (CABI)

1	Qualitative techniques/
2	(interview* or experience or qualitative).ti,ab.
3	Interviews/
4	Ethnography/
5	(phenomenol* or "phenomenological research").ti,ab.
6	local concept*.ti,ab.
7	grounded theory.ti,ab.
8	focus group*.ti,ab.
9	purposive sampl*.ti,ab.
10	("content analys*" or "thematic analys*").ti,ab.
11	Observational method*.ti,ab.
12	Case studies/
13	case stud*.ti,ab.
14	action research.ti,ab.
15	("conversation analys*" or "discourse analys*").ti,ab.
16	interpretive.ti,ab.
17	reveal.ti,ab.
18	1 or 2 or 3 or 4 or 5 or 6 or 7 or 8 or 9 or 10 or 11 or 12 or 13 or 14 or 15 or 16 or 17
19	(Africa or Asia or Caribbean or "West Indies" or "South America" or "Latin America" or "Central America").ti,ab.
20	(Afghanistan or Albania or Algeria or Angola or Antigua or Barbuda or Argentina or Armenia or Armenian or Aruba or Azerbaijan or Bahrain or Bangladesh or Barbados or Benin or Byelarus or Byelorussian or Belarus or Belorussian or Belorussia or Belize or Bhutan or Bolivia or Bosnia or Herzegovina or Hercegovina or Botswana or Brasil or Brazil or Bulgaria or "Burkina Faso" or "Burkina Fasso" or "Upper Volta" or Burundi or Urundi or Cambodia or "Khmer Republic" or Kampuchea or Cameroon or Cameroons or Cameron or Camerons or "Cape Verde" or "Central African Republic" or Chad or China or Colombia or Comoros or "Comoro Islands" or Comores or Mayotte or Congo or Zaire or "Costa Rica" or "Cote d'Ivoire" or "Ivory Coast" or Cuba).ti,ab.
21	(Djibouti or "French Somaliland" or Dominica or "Dominican Republic" or "East Timor" or "East Timur" or "Timor Leste" or Ecuador or Egypt or "United Arab Republic" or "El Salvador" or Eritrea or Ethiopia or Fiji or Gabon or "Gabonese Republic" or Gambia or Gaza or Georgia or Georgian or Ghana or "Gold Coast" or Grenada or Guatemala or Guinea or Guiana or Guyana or Haiti or Honduras or Hungary or India or Maldives or Indonesia or Iran or Iraq or "Isle of Man" or Jamaica or Jordan or Kazakhstan or Kazakh or Kenya or Kiribati or Korea or Kosovo or Kyrgyzstan or

	Kirghizia or "Kyrgyz Republic" or Kirghiz or Kirgizstan or "Lao PDR" or Laos or Lebanon or Lesotho or Basutoland or Liberia or Libya).ti,ab.
22	(Macedonia or Madagascar or "Malagasy Republic" or Malaysia or Malaya or Malay or Sabah or Sarawak or Malawi or Nyasaland or Mali or "Marshall Islands" or Mauritania or Mauritius or "Agalega Islands" or Mexico or Micronesia or "Middle East" or Moldova or Moldovia or Moldovian or Mongolia or Montenegro or Morocco or Ifni or Mozambique or Myanmar or Myanma or Burma or Namibia or Nepal or "Netherlands Antilles" or "New Caledonia" or Nicaragua or Niger or Nigeria or "Northern Mariana Islands" or Oman or Muscat or Pakistan or Palau or Palestine or Panama or Paraguay or Peru or Philippines or Philipines or Phillipines or Phillippines or "Puerto Rico").ti,ab.
23	(Russia or Russian or Rwanda or Ruanda or "Saint Kitts" or "St Kitts" or Nevis or "Saint Lucia" or "St Lucia" or "Saint Vincent" or "St Vincent" or Grenadines or Samoa or "Samoan Islands" or "Navigator Island" or "Navigator Islands" or "Sao Tome" or "Saudi Arabia" or Senegal or Serbia or Montenegro or "Sierra Leone" or "Sri Lanka" or Ceylon or "Solomon Islands" or Somalia or Sudan or Suriname or Surinam or Swaziland or Syria or Tajikistan or Tadzhikistan or Tadjikistan or Tadzhik or Tanzania or Thailand or Togo or "Togolese Republic" or Tonga or Trinidad or Tobago or Tunisia or Turkey or Turkmenistan or Turkmen or Uganda or Ukraine or Uruguay or USSR or "Soviet Union" or "Union of Soviet Socialist Republics" or Uzbekistan or Uzbek or Vanuatu or "New Hebrides" or Venezuela or Vietnam or "Viet Nam" or "West Bank" or Yemen or Yugoslavia or Zambia or Zimbabwe or Rhodesia).ti,ab.
24	((developing or "less* developed" or "under developed" or underdeveloped or "middle income" or "low* income" or underserved or "under served" or deprived or poor*) adj1 (countr* or nation* or population* or world)).ti,ab.
25	((developing or "less* developed" or "under developed" or underdeveloped or "middle income" or "low* income") adj1 (economy or economies)).ti,ab.
26	(low* adj1 (GDP or GNP or "gross domestic" or "gross national")).ti,ab.
27	(low adj3 middle adj3 countr*).ti,ab.
28	(LMIC or LMICs or "third world" or "LAMI country" or "LAMI countries").ti,ab.
29	("transitional country" or "transitional countries").ti,ab.
30	19 or 20 or 21 or 22 or 23 or 24 or 25 or 26 or 27 or 28 or 29
31	Accelerated testing/
32	(("point of care" or POC) and (test* or diagnos*)).ti,ab.
33	poct.ti,ab.
34	((rapid or bedside or bed-side or "near patient") and (test* or diagnos*)).ti,ab.
35	outbreak setting.ti,ab.
36	31 or 32 or 33 or 34 or 35
37	18 and 30 and 36
38	limit 37 to yr="2000 -Current"

Anthropology Plus

1	"Point-of-care testing"
2	("point of care" or POC) and (test* or diagnos*)
	poct
3	(rapid or bedside or bed-side or "near patient") and (test* or diagnos*)
4	"medical innovation*"
5	1 or 2 or 3 or 4 or 5

CINAHL

1	Qualitative studies/
2	Audiorecording/
3	Interviews/
4	interview*
5	Narratives/
6	"experienc*"
7	Attitude/
7	Ethnographic research/
8	ethnograph*
9	Field Studies/
11	Phenomenology/ or Phenomenological research/
12	"local concept*"
13	Grounded Theory/
14	Focus Groups/
15	Purposive Sample/
16	Content Analysis/
17	Thematic Analysis/
18	"thematic analys*"
19	Observational Methods/
20	Participant Observation/
21	"participant observ*"
22	Nonparticipant Observation/
23	Case Studies/
	"case stud*"
24	Action Research/
25	"conversation analys*"
26	Discourse Analysis/
27	S1 or S2 or S3 or S4 or S5 or S6 or S7 or S8 or S9 or S10 or S11 or S12 or S13 or S14 or S15 or S16 or S17 or S18 or S19 or S20 or S21 or S22 or S23 or S24 or S25 or S26 or S27
28	Africa or Asia or Caribbean or "West Indies" or "South America" or "Latin America" or "Central America"
29	Afghanistan OR Albania OR Algeria OR Angola OR Antigua OR Barbuda OR Argentina OR Armenia OR Armenian OR Aruba OR Azerbaijan OR Bahrain OR Bangladesh OR Barbados OR Benin OR Byelarus OR Byelorussian OR Belarus OR Belorussian OR Belorussia OR Belize OR Bhutan OR Bolivia OR Bosnia OR Herzegovina OR Hercegovina OR Botswana OR Brasil OR Brazil OR Bulgaria OR "Burkina Faso" OR "Burkina Fasso" OR "Upper Volta" OR Burundi OR Urundi OR Cambodia OR "Khmer Republic" OR Kampuchea OR Cameroon OR Cameroons OR Cameron OR Camerons OR "Cape Verde" OR "Central African Republic" OR Chad OR China OR Colombia OR Comoros OR "Comoro Islands" OR Comores OR Mayotte OR Congo OR Zaire OR "Costa Rica" OR "Cote d'Ivoire" OR "Ivory Coast" OR Cuba

30	Djibouti or "French Somaliland" or Dominica or "Dominican Republic" or "East Timor" or "East Timur" or "Timor Leste" or Ecuador or Egypt or "United Arab Republic" or "El Salvador" or Eritrea or Ethiopia or Fiji or Gabon or "Gabonese Republic" or Gambia or Gaza or Georgia or Georgian or Ghana or "Gold Coast" or Grenada or Guatemala or Guinea or Guiana or Guyana or Haiti or Honduras or Hungary or India or Maldives or Indonesia or Iran or Iraq or "Isle of Man" or Jamaica or Jordan or Kazakhstan or Kazakh or Kenya or Kiribati or Korea or Kosovo or Kyrgyzstan or Kirghizia or "Kyrgyz Republic" or Kirghiz or Kirgizstan or "Lao PDR" or Laos or Lebanon or Lesotho or Basutoland or Liberia or Libya
31	Macedonia or Madagascar or "Malagasy Republic" or Malaysia or Malaya or Malay or Sabah or Sarawak or Malawi or Nyasaland or Mali or "Marshall Islands" or Mauritania or Mauritius or "Agalega Islands" or Mexico or Micronesia or "Middle East" or Moldova or Moldovia or Moldovian or Mongolia or Montenegro or Morocco or Ifni or Mozambique or Myanmar or Myanma or Burma or Namibia or Nepal or "Netherlands Antilles" or "New Caledonia" or Nicaragua or Niger or Nigeria or "Northern Mariana Islands" or Oman or Muscat or Pakistan or Palau or Palestine or Panama or Paraguay or Peru or Philippines or Philipines or Phillipines or Phillippines or "Puerto Rico"
32	Russia or Russian or Rwanda or Ruanda or "Saint Kitts" or "St Kitts" or Nevis or "Saint Lucia" or "St Lucia" or "Saint Vincent" or "St Vincent" or Grenadines or Samoa or "Samoan Islands" or "Navigator Island" or "Navigator Islands" or "Sao Tome" or "Saudi Arabia" or Senegal or Serbia or Montenegro or "Sierra Leone" or "Sri Lanka" or Ceylon or "Solomon Islands" or Somalia or Sudan or Suriname or Surinam or Swaziland or Syria or Tajikistan or Tadzhikistan or Tadjikistan or Tadzhik or Tanzania or Thailand or Togo or "Togolese Republic" or Tonga or Trinidad or Tobago or Tunisia or Turkey or Turkmenistan or Turkmen or Uganda or Ukraine or Uruguay or USSR or "Soviet Union" or "Union of Soviet Socialist Republics" or Uzbekistan or Uzbek or Vanuatu or "New Hebrides" or Venezuela or Vietnam or "Viet Nam" or "West Bank" or Yemen or Yugoslavia or Zambia or Zimbabwe or Rhodesia)
33	(developing or "less* developed" or "under developed" or underdeveloped or "middle income" or "low* income" or underserved or "under served" or deprived or poor*) N1 (countr* or nation* or population* or world)
34	(developing or "less* developed" or "under developed" or underdeveloped or "middle income" or "low* income") N1 (economy or economies)
35	low* N1 (GDP or GNP or "gross domestic" OR "gross national")
36	low N3 middle N3 countr*
37	LMIC OR LMICs OR "third world" OR "LAMI country" OR "LAMI countries"
38	"transitional country" OR "transitional countries"
39	S29 or S30 or S31 or S32 or S33 or S34 or S35 or S36 or S37 or S38 or S39
40	Point-of-Care Testing/
41	("point of care" or POC) and (test* or diagnos*)
42	Poct
43	(rapid or bedside or bed-side or "near patient") and (test* or diagnos*)
44	("rapid diagnostic test*") or (rdt)
45	"outbreak setting"
46	S41 or S42 or S43 or S44 or S45 or S46
47	S28 and S40 and S47
48	Limiters - Publication Year: 2000-2022

Web of Science

1	TS=("qualitative research")
2	TS=("qualitative method*")

3	TS=("interview*")
4	TS=("ethnograph*")
5	TS=("fieldwork" or "field work")
6	TS=("participant observ*")
7	TS=("phenomenological study")
8	TS=("phenomenological research")
9	TS=("phenomenological approach")
10	TS=("local concept*")
11	TS=("grounded theory")
12	TS=("focus group*")
13	TS=("purposive sampl*")
14	TS=("content analys*")
15	TS=("thematic analys*")
16	TS=("observational method*")
17	TS=("case stud*")
18	TS=("action research")
19	TS=("conversation analys*")
20	TS=("discourse analys*")
21	TS=(interpretive)
22	#1 or #2 or #3 or #4 or #5 or #6 or #7 or #8 or #9 or #10 or #11 or #12 or #13 or #14 or #15 or #16 or #17 or #18 or #19 or #20 or #21
23	TS=(Africa or Asia or Caribbean or "West Indies" or "South America" or "Latin America" or "Central America")
24	TS=(Afghanistan OR Albania OR Algeria OR Angola OR Antigua OR Barbuda OR Argentina OR Armenia OR Armenian OR Aruba OR Azerbaijan OR Bahrain OR Bangladesh OR Barbados OR Benin OR Byelarus OR Byelorussian OR Belarus OR Belorussian OR Belorussia OR Belize OR Bhutan OR Bolivia OR Bosnia OR Herzegovina OR Hercegovina OR Botswana OR Brasil OR Brazil OR Bulgaria OR "Burkina Faso" OR "Burkina Fasso" OR "Upper Volta" OR Burundi OR urundi OR Cambodia OR "Khmer Republic" OR Kampuchea OR Cameroon OR Cameroons OR Cameron OR cameroons OR "Cape Verde" OR "Central African Republic" OR Chad OR China OR Colombia OR Comoros OR "Comoro Islands" OR Comores OR Mayotte OR Congo OR Zaire OR "Costa Rica" OR "Cote d'Ivoire" OR "Ivory Coast" OR Cuba)
25	TS=(Djibouti or "French Somaliland" or Dominica or "Dominican Republic" or "East Timor" or "East Timur" or "Timor Leste" or Ecuador or Egypt or "United Arab Republic" or "El Salvador" or Eritrea or Ethiopia or Fiji or Gabon or "Gabonese Republic" or Gambia or Gaza or Georgia or Georgian or Ghana or "Gold Coast" or Grenada or Guatemala or Guinea or Guiana or Guyana or Haiti or Honduras or Hungary or India or Maldives or Indonesia or Iran or Iraq or "Isle of Man" or Jamaica or Jordan or Kazakhstan or Kazakh or Kenya or Kiribati or Korea or Kosovo or Kyrgyzstan or Kirghizia or "Kyrgyz Republic" or Kirghiz or kyrghizstan or "Lao PDR" or Laos or Lebanon or Lesotho or Basutoland or Liberia or Libya)
26	TS=(Macedonia or Madagascar or "Malagasy Republic" or Malaysia or Malaya or Malay or Sabah or Sarawak or Malawi or Nyasaland or Mali or "Marshall Islands" or Mauritania or Mauritius or "Agalega Islands" or Mexico or Micronesia or "Middle East" or Moldova or moldavia or moldavian or Mongolia or Montenegro or Morocco or Ifni or Mozambique or Myanmar or myanmar or Burma or Namibia or Nepal or "Netherlands Antilles" or "New Caledonia" or Nicaragua or Niger or Nigeria or "Northern Mariana Islands" or Oman or Muscat or Pakistan or Palau or Palestine or Panama or Paraguay or Peru or Philippines or philippines or philippines or philippines or "Puerto Rico")

27	TS=(Russia or Russian or Rwanda or Ruanda or "Saint Kitts" or "St Kitts" or Nevis or "Saint Lucia" or "St Lucia" or "Saint Vincent" or "St Vincent" or Grenadines or Samoa or "Samoan Islands" or "Navigator Island" or "Navigator Islands" or "Sao Tome" or "Saudi Arabia" or Senegal or Serbia or Montenegro or "Sierra Leone" or "Sri Lanka" or Ceylon or "Solomon Islands" or Somalia or Sudan or Suriname or Surinam or Swaziland or Syria or Tajikistan or Tadzhikistan or Tadjikistan or tadzhika or Tanzania or Thailand or Togo or "Togolese Republic" or Tonga or Trinidad or Tobago or Tunisia or Turkey or Turkmenistan or Turkmen or Uganda or Ukraine or Uruguay or USSR or "Soviet Union" or "Union of Soviet Socialist Republics" or Uzbekistan or Uzbek or Vanuatu or "New Hebrides" or Venezuela or Vietnam or "Viet Nam" or "West Bank" or Yemen or Yugoslavia or Zambia or Zimbabwe or Rhodesia)
28	TS=((developing or "less* developed" or "under developed" or underdeveloped or "middle income" or "low* income" or underserved or "under served" or deprived or poor*) NEAR/0 (countr* or nation* or population* or world))
29	TS=((developing or "less* developed" or "under developed" or underdeveloped or "middle income" or "low* income") NEAR/1 (economy or economies))
30	TS=(low* NEAR/0 (GDP or GNP or "gross domestic" OR "gross national"))
31	TS=(low NEAR/3 middle NEAR/3 countr*)
32	TS=(LMIC OR LMICs OR "third world" OR "LAMI country" OR "LAMI countries")
33	TS=("transitional country" OR "transitional countries")
34	#23 or #24 or #25 or #26 or #27 or #28 or #29 or #30 or #31 or #32 or #33
35	TS=((("point of care" or "point-of-care") or POC) AND (test* or diagnos* or system))
36	TS=poct
37	TS=(((rapid or bedside or bed-side or "near patient") and (test* or diagnos*)))
38	TS=("outbreak setting")
39	#35 or #36 or #36 or #38
40	#22 and #34 and #39
41	#22 and #34 and #39 and 2023 or 2022 or 2021 or 2020 or 2019 or 2018 or 2017 or 2023 or 2022 or 2021 or 2020 or 2019 or 2018 or 2017 or 2016 or 2015 or 2014 or 2013 or 2012 or 2011 or 2010 or 2009 or 2008 or 2007 or 2006 or 2005 or 2004 or 2003 or 2002 or 2001 or 2000 (Publication Years)

Scopus

41	((TITLE-ABS-KEY ("qualitative research")) OR (TITLE-ABS-KEY ("qualitative method*")) OR (TITLE-ABS-KEY ("interview*")) OR (TITLE-ABS-KEY ("ethnograph*")) OR (TITLE-ABS-KEY ("fieldwork" OR "field work")) OR (TITLE-ABS-KEY ("participant observ*")) OR (TITLE-ABS-KEY ("phenomenological study")) OR (TITLE-ABS-KEY ("phenomenological research")) OR (TITLE-ABS-KEY ("phenomenological approach")) OR (TITLE-ABS-KEY ("local concept*")) OR (TITLE-ABS-KEY ("grounded theory")) OR (TITLE-ABS-KEY ("focus group*")) OR (TITLE-ABS-KEY ("purposive sampl*")) OR (TITLE-ABS-KEY ("content analys*")) OR (TITLE-ABS-KEY ("thematic analys*")) OR (TITLE-ABS-KEY ("observational method*")) OR (TITLE-ABS-KEY ("case stud*")) OR (TITLE-ABS-KEY ("action research")) OR (TITLE-ABS-KEY ("conversation analys*")) OR (TITLE-ABS-KEY ("discourse analys*")) OR (TITLE-ABS-KEY (interpretive))) AND (#23 OR #24 OR #25 OR #26 OR #27 OR #28 OR #29 OR #30 OR #31 OR #32 OR #33) AND ((TITLE-ABS-KEY ((("point of care" OR "point-of-care") OR poc) AND (test* OR diagnos* OR system))) OR (TITLE-ABS-KEY (poct)) OR (TITLE-ABS-KEY (poct)) OR (TITLE-ABS-KEY ("outbreak setting"))) AND (LIMIT-TO (PUBYEAR , 2022) OR LIMIT-TO (PUBYEAR , 2021) OR LIMIT-TO (PUBYEAR , 2020) OR LIMIT-TO (PUBYEAR , 2019) OR LIMIT-TO (PUBYEAR , 2018) OR LIMIT-TO (PUBYEAR , 2017) OR LIMIT-TO (PUBYEAR , 2016) OR LIMIT-TO (PUBYEAR , 2015) OR LIMIT-TO (PUBYEAR , 2014) OR LIMIT-TO (PUBYEAR , 2013) OR LIMIT-TO (PUBYEAR , 2012) OR LIMIT-TO (PUBYEAR , 2011) OR LIMIT-TO (PUBYEAR , 2010) OR LIMIT-TO (PUBYEAR , 2009) OR LIMIT-TO (PUBYEAR , 2008) OR LIMIT-TO (PUBYEAR , 2007) OR LIMIT-TO (PUBYEAR , 2006) OR LIMIT-TO (PUBYEAR , 2005) OR LIMIT-TO (PUBYEAR , 2004) OR LIMIT-TO (PUBYEAR , 2003) OR LIMIT-TO (PUBYEAR , 2002) OR LIMIT-TO (PUBYEAR , 2001) OR LIMIT-TO (PUBYEAR , 2000))
40	((TITLE-ABS-KEY ("qualitative research")) OR (TITLE-ABS-KEY ("qualitative method*")) OR (TITLE-ABS-KEY ("interview*")) OR (TITLE-ABS-KEY ("ethnograph*")) OR (TITLE-ABS-KEY ("fieldwork" OR "field work")) OR (TITLE-ABS-KEY ("participant observ*")) OR (TITLE-ABS-KEY ("phenomenological study")) OR (TITLE-ABS-KEY ("phenomenological research")) OR (TITLE-ABS-KEY ("phenomenological approach")) OR (TITLE-ABS-KEY ("local concept*")) OR (TITLE-ABS-KEY ("grounded theory")) OR (TITLE-ABS-KEY ("focus group*")) OR (TITLE-ABS-KEY ("purposive sampl*")) OR (TITLE-ABS-KEY ("content analys*")) OR (TITLE-ABS-KEY ("thematic analys*")) OR (TITLE-ABS-KEY ("observational method*")) OR (TITLE-ABS-KEY ("case stud*")) OR (TITLE-ABS-KEY ("action research")) OR (TITLE-ABS-KEY ("conversation analys*")) OR (TITLE-ABS-KEY ("discourse analys*")) OR (TITLE-ABS-KEY (interpretive))) AND (#23 OR #24 OR #25 OR #26 OR #27 OR #28 OR #29 OR #30 OR #31 OR #32 OR #33) AND ((TITLE-ABS-KEY ((("point of care" OR "point-of-care") OR poc) AND (test* OR diagnos* OR system))) OR (TITLE-ABS-KEY (poct)) OR (TITLE-ABS-KEY (poct)) OR (TITLE-ABS-KEY ("outbreak setting"))) ...View More
39	(TITLE-ABS-KEY ((("point of care" OR "point-of-care") OR poc) AND (test* OR diagnos* OR system))) OR (TITLE-ABS-KEY (poct)) OR (TITLE-ABS-KEY (poct)) OR (TITLE-ABS-KEY ("outbreak setting"))
38	TITLE-ABS-KEY ("outbreak setting")

37	TITLE-ABS-KEY (((rapid OR bedside OR bed-side OR "near patient") AND (test* OR diagnos*)))
36	TITLE-ABS-KEY (poct)
35	TITLE-ABS-KEY ((("point of care" OR "point-of-care") OR poc) AND (test* OR diagnos* OR system))
34	#23 OR #24 OR #25 OR #26 OR #27 OR #28 OR #29 OR #30 OR #31 OR #32 OR #33
33	TITLE-ABS-KEY ("transitional country" OR "transitional countries")
32	TITLE-ABS-KEY (lmic OR lmics OR "third world" OR "LAMI country" OR "LAMI countries")
31	TITLE-ABS-KEY (low AND near/3 AND middle AND near/3 AND countr*)
30	TITLE-ABS-KEY (low* AND near/0 (gdp OR gnp OR "gross domestic" OR "gross national"))
29	TITLE-ABS-KEY ((developing OR "less* developed" OR "under developed" OR underdeveloped OR "middle income" OR "low* income") near/1 (economy OR economies))
28	TITLE-ABS-KEY ((developing OR "less* developed" OR "under developed" OR underdeveloped OR "middle income" OR "low* income" OR underserved OR "under served" OR deprived OR poor*) near/0 (countr* OR nation* OR population* OR world))
27	TITLE-ABS-KEY (russia OR russian OR rwanda OR ruanda OR "Saint Kitts" OR "St Kitts" OR nevis OR "Saint Lucia" OR "St Lucia" OR "Saint Vincent" OR "St Vincent" OR grenadines OR samoa OR "Samoan Islands" OR "Navigator Island" OR "Navigator Islands" OR "Sao Tome" OR "Saudi Arabia" OR senegal OR serbia OR montenegro OR "Sierra Leone" OR "Sri Lanka" OR ceylon OR "Solomon Islands" OR somalia OR sudan OR suriname OR surinam OR swaziland OR syria OR tajikistan OR tadzhikistan OR tadjikistan OR tadzhika OR tanzania OR thailand OR togo OR "Togolese Republic" OR tonga OR trinidad OR tobago OR tunisia OR turkey OR turkmenistan OR turkmen OR uganda OR ukraine OR uruguay OR ussr OR "Soviet Union" OR "Union of Soviet Socialist Republics" OR uzbekistan OR uzbek OR vanuatu OR "New Hebrides" OR venezuela OR vietnam OR "Viet Nam" OR "West Bank" OR yemen OR yugoslavia OR zambia OR zimbabwe OR rhodesia)
26	TITLE-ABS-KEY (macedonia OR madagascar OR "Malagasy Republic" OR malaysia OR malaya OR malay OR sabah OR sarawak OR malawi OR nyasaland OR mali OR "Marshall Islands" OR mauritania OR mauritius OR "Agalega Islands" OR mexico OR micronesia OR "Middle East" OR moldova OR moldavia OR moldavian OR mongolia OR montenegro OR morocco OR ifni OR mozambique OR myanmar OR myanmar OR burma OR namibia OR nepal OR " Netherlands Antilles" OR "New Caledonia" OR nicaragua OR niger OR nigeria OR "Northern Mariana Islands" OR oman OR muscat OR pakistan OR palau OR palestine OR panama OR paraguay OR peru OR philippines OR philippines OR philippines OR philippines OR "Puerto Rico")
25	TITLE-ABS-KEY (djibouti OR "French Somaliland" OR dominica OR "Dominican Republic" OR "East Timor" OR "East Timur" OR "Timor Leste" OR ecuador OR egypt OR "United Arab Republic" OR "El Salvador" OR eritrea OR ethiopia OR fiji OR gabon OR "Gabonese

	Republic" OR gambia OR gaza OR georgia OR georgian OR ghana OR "Gold Coast" OR grenada OR guatemala OR guinea OR guiana OR guyana OR haiti OR honduras OR hungary OR india OR maldives OR indonesia OR iran OR iraq OR "Isle of Man" OR jamaica OR jordan OR kazakhstan OR kazakh OR kenya OR kiribati OR korea OR kosovo OR kyrgyzstan OR kirghizia OR "Kyrgyz Republic" OR kirghiz OR kyrghizstan OR "Lao PDR" OR laos OR lebanon OR lesotho OR basutoland OR liberia OR libya)
24	TITLE-ABS-KEY (afghanistan OR albania OR algeria OR angola OR antigua OR barbuda OR argentina OR armenia OR armenian OR aruba OR azerbaijan OR bahrain OR bangladesh OR barbados OR benin OR byelarus OR byelorussian OR belarus OR belorussian OR belorussia OR belize OR bhutan OR bolivia OR bosnia OR herzegovina OR hercegovina OR botswana OR brasil OR brazil OR bulgaria OR "Burkina Faso" OR "Burkina Fasso" OR "Upper Volta" OR burundi OR urundi OR cambodia OR "Khmer Republic" OR kampuchea OR cameroon OR cameroons OR cameron OR cameroons OR "Cape Verde" OR "Central African Republic" OR chad OR china OR colombia OR comoros OR "Comoro Islands" OR comores OR mayotte OR congo OR zaire OR "Costa Rica" OR "Cote d'Ivoire" OR "Ivory Coast" OR cuba)
23	TITLE-ABS-KEY (africa OR asia OR caribbean OR "West Indies" OR "South America" OR "Latin America" OR "Central America")
22	(TITLE-ABS-KEY ("qualitative research")) OR (TITLE-ABS-KEY ("qualitative method*")) OR (TITLE-ABS-KEY ("interview*")) OR (TITLE-ABS-KEY ("ethnograph*")) OR (TITLE-ABS-KEY ("fieldwork" OR "field work")) OR (TITLE-ABS-KEY ("participant observ*")) OR (TITLE-ABS-KEY ("phenomenological study")) OR (TITLE-ABS-KEY ("phenomenological research")) OR (TITLE-ABS-KEY ("phenomenological approach")) OR (TITLE-ABS-KEY ("local concept*")) OR (TITLE-ABS-KEY ("grounded theory")) OR (TITLE-ABS-KEY ("focus group*")) OR (TITLE-ABS-KEY ("purposive sampl*")) OR (TITLE-ABS-KEY ("content analys*")) OR (TITLE-ABS-KEY ("thematic analys*")) OR (TITLE-ABS-KEY ("observational method*")) OR (TITLE-ABS-KEY ("case stud*")) OR (TITLE-ABS-KEY ("action research")) OR (TITLE-ABS-KEY ("conversation analys*")) OR (TITLE-ABS-KEY ("discourse analys*")) OR (TITLE-ABS-KEY (interpretive))
21	TITLE-ABS-KEY (interpretive)
20	TITLE-ABS-KEY ("discourse analys*")
19	TITLE-ABS-KEY ("conversation analys*")
18	TITLE-ABS-KEY ("action research")
17	TITLE-ABS-KEY ("case stud*")
16	TITLE-ABS-KEY ("observational method*")
15	TITLE-ABS-KEY ("thematic analys*")
14	TITLE-ABS-KEY ("content analys*")
13	TITLE-ABS-KEY ("purposive sampl*")
12	TITLE-ABS-KEY ("focus group*")
11	TITLE-ABS-KEY ("grounded theory")
10	TITLE-ABS-KEY ("local concept*")
9	TITLE-ABS-KEY ("phenomenological approach")
8	TITLE-ABS-KEY ("phenomenological research")
7	TITLE-ABS-KEY ("phenomenological study")
6	TITLE-ABS-KEY ("participant observ*")
5	TITLE-ABS-KEY ("fieldwork" OR "field work")
4	TITLE-ABS-KEY ("ethnograph*")

3	TITLE-ABS-KEY ("interview*")
2	TITLE-ABS-KEY ("qualitative method*")
1	TITLE-ABS-KEY ("qualitative research")

Global Index Medicus-World Health Organization

	tw:((tw:(qualitative analysis/)) OR (tw:(qualitative research/)) OR (tw:(qualitative method*)) OR (tw:(interview/)) OR (tw:(interview*)) OR (tw:(personal narrative/)) OR (tw:(anthropology/)) OR (tw:(sociology/)) OR (tw:(ethnograph*)) OR (tw:((phenomenol* OR "phenomenological research"))) OR (tw:("local concept*")) OR (tw:("grounded theory")) OR (tw:("focus group*")) OR (tw:("purposive sampl*")) OR (tw:(("content analys*" OR "thematic analys*"))) OR (tw:("observational method*")) OR (tw:("participant observation*")) OR (tw:("case stud*")) OR (tw:("action research")) OR (tw:(("conversation analys*" OR "discourse analys*"))))
AND	tw:((tw:(tw:(africa OR asia OR caribbean OR "West Indies" OR "South America" OR "Latin America" OR "Central America"))) OR (tw:(tw:(afghanistan OR albania OR algeria OR angola OR antigua OR barbuda OR argentina OR armenia OR armenian OR aruba OR azerbaijan OR bahrain OR bangladesh OR barbados OR benin OR byelarus OR byelorussian OR belarus OR belorussian OR belorussia OR belize OR bhutan OR bolivia OR bosnia OR herzegovina OR hercegovina OR botswana OR brasil OR brazil OR bulgaria OR "Burkina Faso" OR "Burkina Fasso" OR "Upper Volta" OR burundi OR urundi OR cambodia OR "Khmer Republic" OR kampuchea OR cameroon OR cameroons OR cameron OR camerons OR "Cape Verde" OR "Central African Republic" OR chad OR china OR colombia OR comoros OR "Comoro Islands" OR comores OR mayotte OR congo OR zaire OR "Costa Rica" OR "Cote d'Ivoire" OR "Ivory Coast" OR cuba))) OR (tw:(tw:(djibouti OR "French Somaliland" OR dominica OR "Dominican Republic" OR "East Timor" OR "East Timur" OR "Timor Leste" OR ecuador OR egypt OR "United Arab Republic" OR "El Salvador" OR eritrea OR ethiopia OR fiji OR gabon OR "Gabonese Republic" OR gambia OR gaza OR georgia OR georgian OR ghana OR "Gold Coast" OR grenada OR guatemala OR guinea OR guiana OR guyana OR haiti OR honduras OR hungary OR india OR maldives OR indonesia OR iran OR iraq OR "Isle of Man" OR jamaica OR jordan OR kazakhstan OR kazakh OR kenya OR kiribati OR korea OR kosovo OR kyrgyzstan OR kirghizia OR "Kyrgyz Republic" OR kirghiz OR kirgizstan OR "Lao PDR" OR laos OR lebanon OR lesotho OR basutoland OR liberia OR libya))) OR (tw:(tw:(macedonia OR madagascar OR "Malagasy Republic" OR malaysia OR malaya OR malay OR sabah OR sarawak OR malawi OR nyasaland OR mali OR "Marshall Islands" OR mauritania OR mauritius OR "Agalega Islands" OR mexico OR micronesia OR "Middle East" OR moldova OR moldovia OR moldovian OR mongolia OR montenegro OR morocco OR ifni OR mozambique OR myanmar OR myanma OR burma OR namibia OR nepal OR "Netherlands Antilles" OR "New Caledonia" OR nicaragua OR niger OR nigeria OR "Northern Mariana Islands" OR oman OR muscat OR pakistan OR palau OR palestine OR panama OR paraguay OR peru OR philippines OR philipines OR phillipines OR phillippines OR "Puerto Rico"))) OR (tw:(tw:(russia OR russian OR rwanda OR ruanda OR "Saint Kitts" OR "St Kitts" OR nevis OR "Saint Lucia" OR "St Lucia" OR "Saint Vincent" OR "St Vincent" OR grenadines OR samoa OR "Samoan Islands" OR "Navigator Island" OR "Navigator Islands" OR "Sao Tome" OR "Saudi Arabia" OR senegal OR serbia OR montenegro OR "Sierra Leone" OR "Sri Lanka" OR ceylon OR "Solomon Islands" OR somalia OR sudan OR suriname OR surinam OR swaziland OR syria OR tajikistan OR tadzhikistan OR tadjikistan OR tadzhik OR tanzania OR thailand OR togo OR "Togolese Republic" OR tonga OR trinidad OR tobago OR tunisia OR turkey OR turkmenistan OR turkmen OR uganda OR ukraine OR uruguay OR ussr OR "Soviet Union" OR "Union of Soviet Socialist Republics" OR uzbekistan OR uzbek OR vanuatu OR "New Hebrides" OR venezuela

	OR vietnam OR "Viet Nam" OR "West Bank" OR yemen OR yugoslavia OR zambia OR zimbabwe OR rhodesia))) OR (tw:(tw:((developing OR "less* developed" OR "under developed" OR underdeveloped OR "middle income" OR "low* income" OR underserved OR "under served" OR deprived OR poor*) AND (countr* OR nation* OR population* OR world)))) OR (tw:(tw:(lmic OR lmics OR "third world" OR "LAMI country" OR "LAMI countries"))))
	tw:((tw:(point-of-care system/)) OR (tw:(point-of-care testing/)) OR (tw:(tw:(("point of care" OR poc) AND (test* OR diagnos*)))) OR (tw:(tw:poct)) OR (tw:(tw:((rapid) AND (test* OR diagnos*) OR (rdt)))))
