# Supplementary material for: Is qualitative social research in global health fulfilling its potential?: a systematic evidence mapping of research on point-of-care testing in low- and middle-income contexts
Source: BMC Health Serv Res. 2024 Feb 7;24:172. doi: 10.1186/s12913-024-10645-5 (PMC10848363; doi:10.1186/s12913-024-10645-5)
Supplement: Supplementary file 2 — Additional file 2. Selection criteria. [file 12913_2024_10645_MOESM2_ESM.pdf]

Additional File 2: Selection criteria

SM Table 1: Inclusion/exclusion criteria

Category	Inclusion	Exclusion
Publication date	After 2000	Before 2000
Type of evidence	Published studies; research studies; empirical studies; grey literature in the form of theses and dissertations	Literature reviews; comment/opinion articles; pre-prints; grey literature in the form of reports and policy documents; abstracts and conference proceedings
Type of study (i.e. study design, methodology)	Qualitative studies (action research, case study analysis, discourse analysis, ethnography, focus group discussion-based, grounded theory, interview-based, narrative analysis, observation, participant observation, phenomenology); mixed-method studies where qualitative evidence is epistemologically distinct from and reported separately to quantitative evidence.	Quantitative studies; survey-based studies with open-ended questions; qualitative process evaluations; realist evaluations; mixed-method studies where qualitative evidence epistemologically resembles and/or is reported alongside quantitative evidence; study protocols
Environment	LMICs as context of deployment and use; research and development, regulatory, and policymaking settings based in high-income or LMIC environments that pertain to POCTs for use in LMICs	High-income countries as context of deployment and use; research and development, regulatory, and policymaking settings that pertain to POCTs for use in high-income countries primarily
Test format	In vitro POCTs (e.g., lateral flow immunoassays, molecular tests, nucleic acid amplification tests)	In vivo POCTs; urine-based pregnancy confirmation tests
Setting	POCT research and development settings; regulatory, policymaking, and programming settings; secondary health system settings; primary health system settings; community settings; non-formal health settings (e.g., drug shops); domestic settings	Studies in which the primary setting was laboratories
Perspective	Any stakeholder perspective, e.g., those of POCT researchers and developers (scientists, academics), commercial actors (industry, manufacturers, salespeople) regulators, policymakers, health managers, formal health service providers, informal health service providers, patients	Studies in which lab technician perspectives were primarily taken into account
Phenomena	Any social phenomena related to POCTs, either as a focus or peripherally	Social phenomena unrelated to POCTs, phenomena unrelated to social aspects

Notes on exclusions: Urine-based pregnancy confirmation tests: While urine-based pregnancy confirmation tests are included among the POCTs recommended by the EDL, we excluded papers focused on these tests. First, these tests were developed in the 1970s and therefore have a longer and separate history relative to the POCTs were included in our study. Second, the tests we included are all designed to detect health disorders. Pregnancy, while it may demand particular forms of care, does not in itself represent a health disorder.

Laboratories: While POCTs are generally designed for settings outside the laboratory, in some cases they may be used primarily in laboratories. AS our primary interest was to understand the

decentralisation of tests, we excluded papers that reported on studies carried out primarily in laboratory settings.
