# Supplementary figures and images for: Is qualitative social research in global health fulfilling its potential?: a systematic evidence mapping of research on point-of-care testing in low- and middle-income contexts
Source: BMC Health Serv Res. 2024 Feb 7;24:172. doi: 10.1186/s12913-024-10645-5 (PMC10848363; doi:10.1186/s12913-024-10645-5)

Additional File 3: Reported research methods

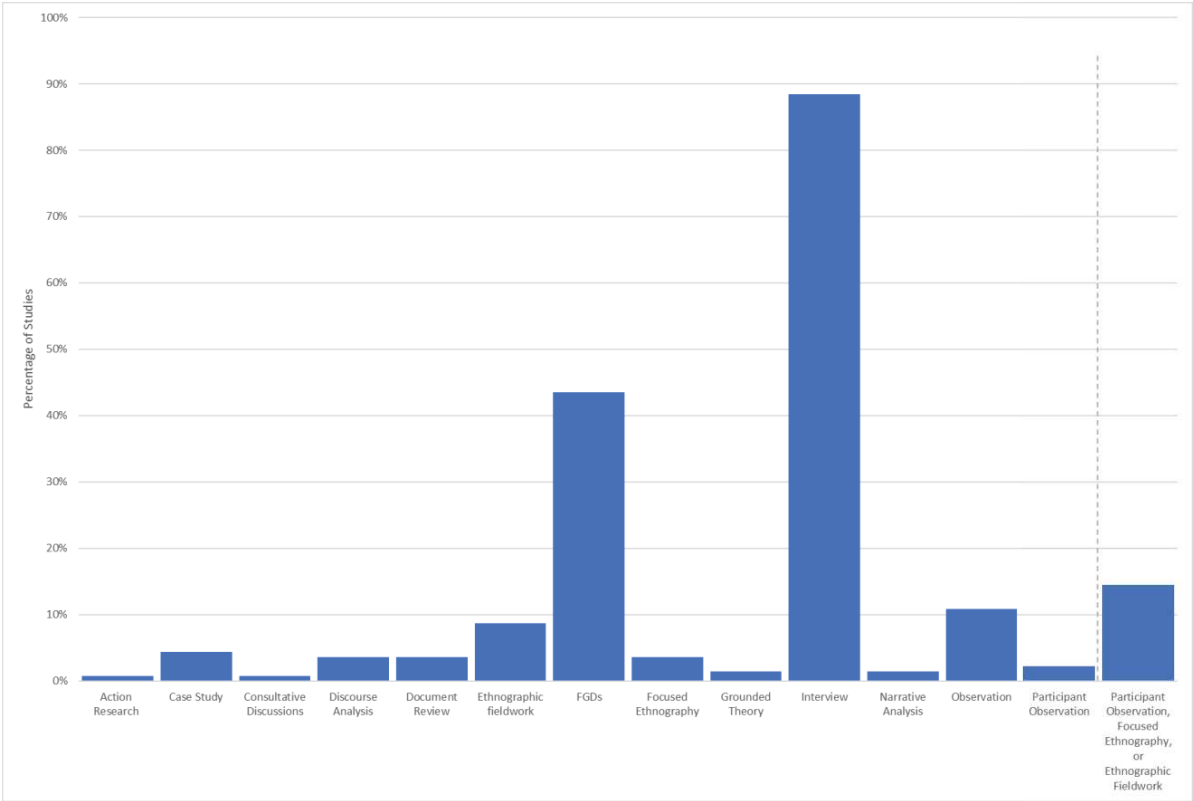

Supplement: Supplementary file 3 — Additional file 3. Reported research methods. [file 12913_2024_10645_MOESM3_ESM.pdf]

Additional File 4: Chronology of reported health conditions

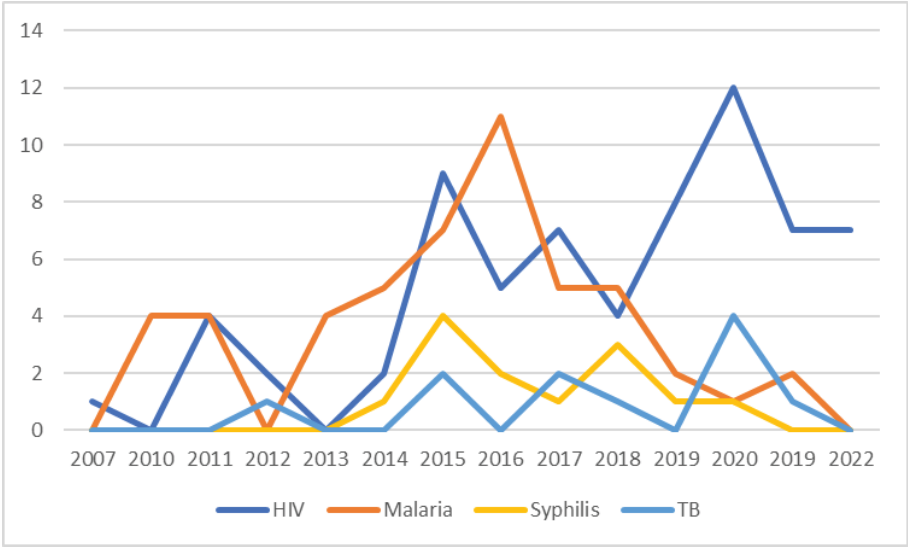

Supplement: Supplementary file 4 — Additional file 4. Chronology of reported health conditions. [file 12913_2024_10645_MOESM4_ESM.pdf]

Additional File 5: Chronology of HIV and malaria-reporting papers

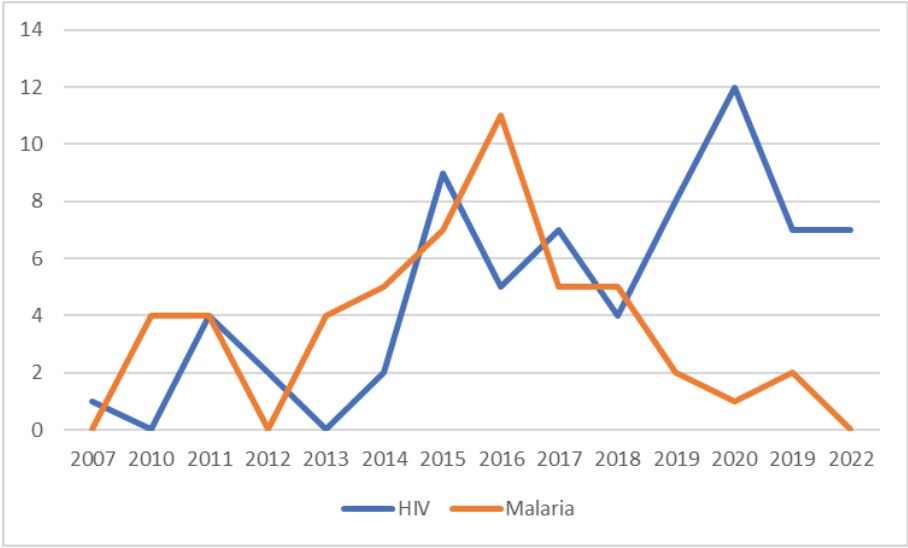

Supplement: Supplementary file 5 — Additional file 5. Chronology of HIV and malaria-reporting papers. [file 12913_2024_10645_MOESM5_ESM.pdf]

Additional File 6: Reported test format

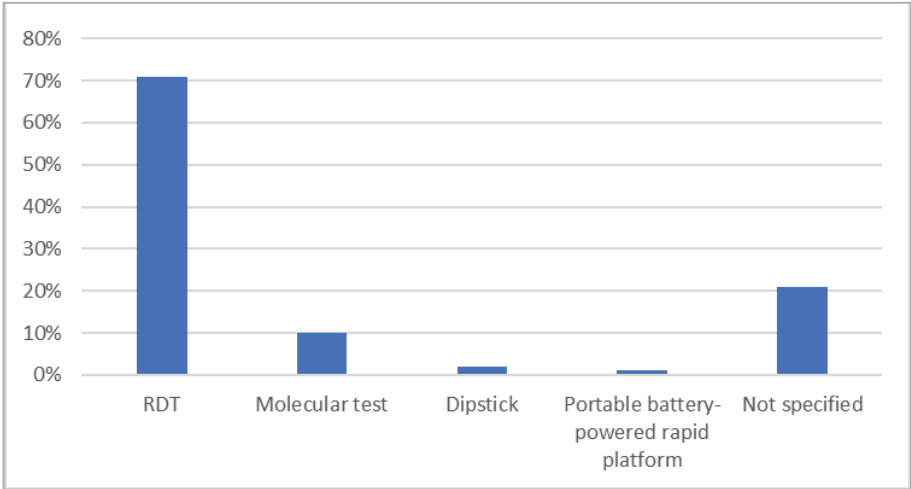

Supplement: Supplementary file 6 — Additional file 6. Reported test format. [file 12913_2024_10645_MOESM6_ESM.pdf]

Additional File 7: Study setting

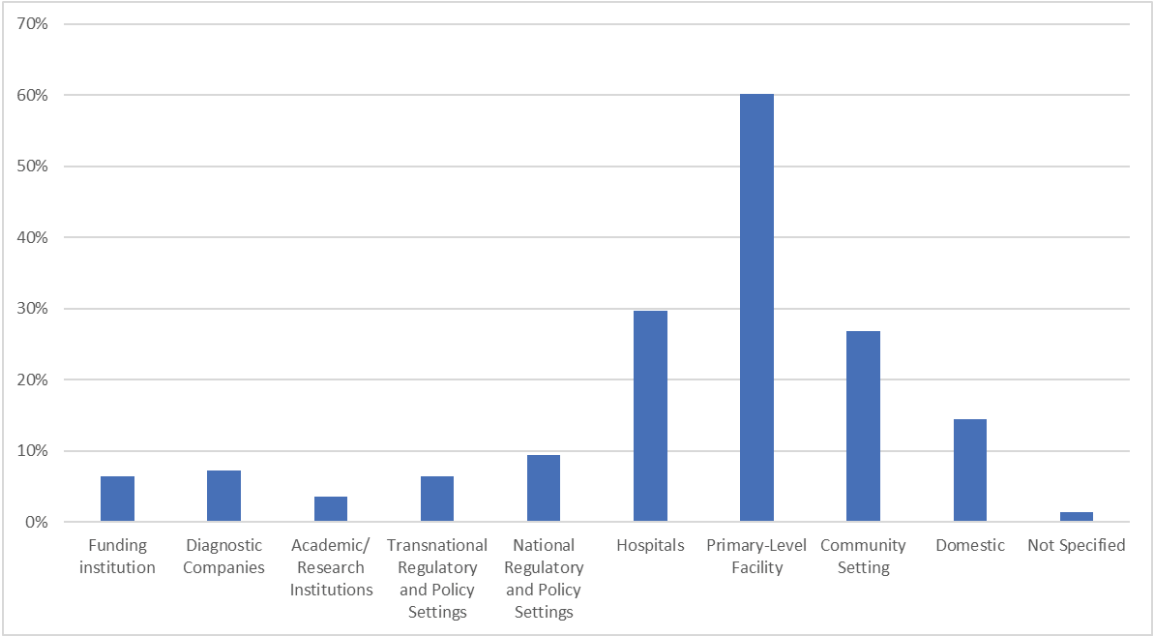

Supplement: Supplementary file 7 — Additional file 7. Study setting. [file 12913_2024_10645_MOESM7_ESM.pdf]

Additional File 8: Reported settings as public or private

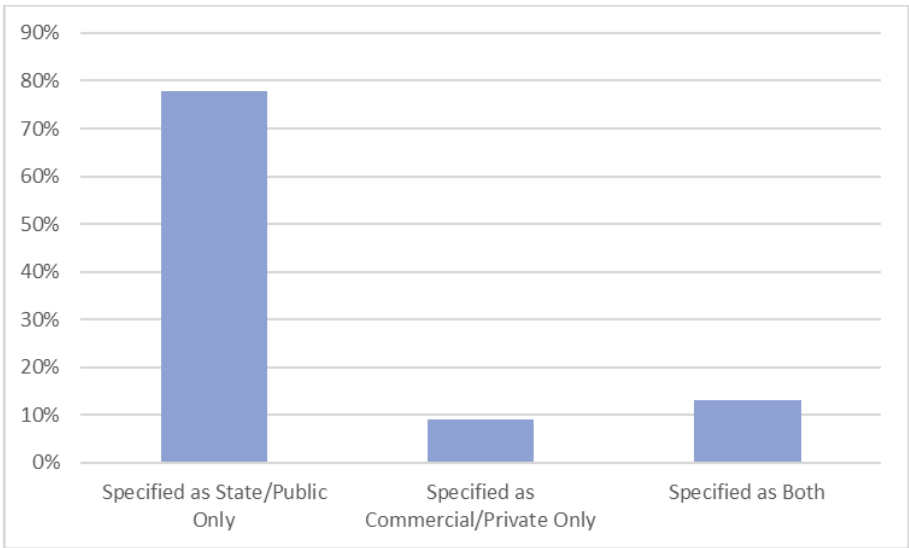

Supplement: Supplementary file 8 — Additional file 8. Reported settings as public or private. [file 12913_2024_10645_MOESM8_ESM.pdf]
