# Supplementary material for: Is qualitative social research in global health fulfilling its potential?: a systematic evidence mapping of research on point-of-care testing in low- and middle-income contexts
Source: BMC Health Serv Res. 2024 Feb 7;24:172. doi: 10.1186/s12913-024-10645-5 (PMC10848363; doi:10.1186/s12913-024-10645-5)
Supplement: Supplementary file 9 — Additional file 9. Reported perspectives. [file 12913_2024_10645_MOESM9_ESM.pdf]

Additional File 9: Reported perspectives

Category	No.	%	Papers
Funders	8	6%	25,35,36,37,38,62,64,118
Researchers and developers	11	8%	25,29,35,36,37,38,62,64,66,78,127
Industry	15	11%	25,29,30,31,34,35,36,37,38,61,62,64,118, 127,128
Regulators	6	4%	
Transnational	5	4%	35,36,38,62,64
National	2	1%	29,62
Policymakers	18	13%	
Transnational	8	6%	34,35,36,37,38,62,64,88,118
National	13	9%	29,30,37,62,64,66,71,75,81,82,88,118,119 , 127
Formal health workforce	103	75%	
Health managers	28	20%	15, 16, 19, 20, 21, 27, 32, 34, 35, 41, 42, 43, 61, 67, 73, 75, 78, 79, 84, 91, 98, 108, 110, 112, 113, 119, 136, 138
Facility-based health care providers	99	72%	1, 2, 4, 5, 6, 9, 10, 13, 14, 15, 17, 18, 19, 20, 21, 22, 26, 27, 28, 30, 31, 32, 33, 34, 35, 36, 38, 39, 40, 41, 42, 44, 45, 46, 48, 49, 50, 51, 52, 53, 55, 56, 57, 58, 60, 61, 65, 66, 67, 71, 72, 73, 75, 76, 78, 79, 80, 81, 82, 83, 84, 85, 86, 88, 89, 91, 92, 94, 95, 96, 97, 98, 100, 101, 104, 105, 106, 108, 109, 110, 112, 113, 114, 115, 119, 121, 122, 123, 124, 125, 126, 129, 130, 131, 133, 135, 136, 137, 138
Community health workers	20	14%	15, 17, 21, 28, 30, 32, 33, 34, 35, 58, 61, 73, 74, 84, 91, 98, 111, 113, 121, 136
Informal health workforce	19	14%	
Healers	3	2%	30, 32, 102
Drug retailers	10	7%	10, 20, 52, 53, 79, 80, 91, 107, 117, 124
School teachers	2	1%	82, 84
Project implementation staff/administrators	10	7%	
Programme/project implementation staff	8	6%	29, 30, 37, 86, 88, 92, 110, 113
Administrative staff	2	1%	19, 99
Advocates	1	1%	86
Civil society	11	8%	
Civil society-Unspecified	5	4%	35, 36, 37, 38, 84
NGOs	6	4%	75, 81, 82, 91, 118, 127
Counsellors	8	6%	
Counsellors	7	5%	9, 46, 85, 110, 119, 121, 122
Peer-counsellors	2	1%	110, 129

Patients	41	30%	1, 3, 7, 10, 12, 19, 23, 24, 27, 30, 31, 32, 33, 34, 42, 44, 45, 46, 49, 50, 51, 54, 55, 56, 57, 59, 70, 76, 82, 83, 87, 93, 97, 99, 101, 102, 112, 114, 116, 124, 135
Family members	21	15%	
Caregivers	18	13%	3, 22, 58, 60, 63, 65, 67, 74, 82, 84, 91, 103, 108, 121, 123, 130, 133, 134
Male partners	2	1%	7, 23
Groups identified as "at risk" or "vulnerable"	12	9%	
Female sex workers	3	2%	8, 17, 132
Men who have sex with men	6	4%	11, 17, 69, 96, 101, 132
Trans women	1	1%	132
People who inject drugs	4	3%	47, 77, 120, 132
Women who use PReP	1	1%	122
Adolescents	1	1%	103
Public/community	15	11%	17, 20, 28, 58, 63, 68, 74, 84, 90, 91, 93, 94, 98, 111, 131

Note on community: Descriptors provided for participants labelled as ‘community members’ were vague and inconsistently. Some studies used demographic descriptors to characterise the group they referred to as ‘community’, for example age and sex, differentiating between men, women, adolescents and adults. However, frequently descriptors relating to sex were often conflated with familial relations and caregiver-related responsibilities including 'mothers', 'fathers' and 'caregivers' as participant categories (Ansah et al., 2013; Chandler et al., 2011; Katirayi et al., 2020; Mukanga et al.; 2010; O'Neil et al., 2016). Common social categories used were opinion and community leaders which were often further broken out into sub-groups including church leaders, political leaders, and activists (Chandler et al., 2011; Diggle et al., 2014; Jegede et al., 2016; Katirayi et al., 2020; Macharia et al., 2020; Mukanga et al., 2010; Nsagha et al., 2011; Shuford et al., 2016). For some studies vagueness in describing the community was purposeful as there were several efforts made within the study to ensure a truly random population sample with no unique descriptors or repeated demographic categories (Bwalya et al., 2020; O'Neil et al., 2016).
