# Supplementary material for: Is qualitative social research in global health fulfilling its potential?: a systematic evidence mapping of research on point-of-care testing in low- and middle-income contexts
Source: BMC Health Serv Res. 2024 Feb 7;24:172. doi: 10.1186/s12913-024-10645-5 (PMC10848363; doi:10.1186/s12913-024-10645-5)
Supplement: Supplementary file 10 — Additional file 10. Summary of included papers. [file 12913_2024_10645_MOESM10_ESM.pdf]

Additional File 10: Summary of included papers

#	Author	Year	Journal	Title	Focus
1	Altaras, R.; Nuwa, A.; Agaba, B.; Streat, E.; Tibenderana, J. K.; Martin, S.; Strachan, C. E.	2016	PLoS ONE	How Do Patients and Health Workers Interact around Malaria Rapid Diagnostic Testing, and How Are the Tests Experienced by Patients in Practice? A Qualitative Study in Western Uganda	To explore how healthcare providers and patients in Uganda interact and engage throughout the diagnostic and treatment process for malaria when presenting with fever during routine outpatient visits, and how the rapid diagnostic testing for malaria (mRDTs) is experienced by patients in practice
2	Altaras, R.; Nuwa, A.; Agaba, B.; Streat, E.; Tibenderana, J. K.; Strachan, C. E.	2016	Malaria journal	Why do health workers give anti-malarials to patients with negative rapid test results? A qualitative study at rural health facilities in western Uganda	To explore the influences on provider decision-making in Uganda regarding the prescription of anti-malarials following a negative RDT result
3	Ansah, E. K.; Reynolds, J.; Akanpigbiam, S.; Whitty, C. J. M.; Chandler, C. I. R.	2013	Malaria Journal	"Even if the test result is negative, they should be able to tell us what is wrong with us": a qualitative study of patient expectations of rapid diagnostic tests for malaria	To explore patient and caregiver perceptions and experiences of malaria RDTs in Ghana; to better understand the complexities surrounding the role of mRDTs in appropriate treatment of fever and to understand patient perspectives on mRDTs beyond notions of acceptability
4	Anticona Huaynate, C. F.; Pajuelo Travezano, M. J.; Correa, M.; Malpartida, H. M.; Oberhelman, R.; Murphy, L. L.; Paz-Soldan, V. A.	2015	BMC Health Services Research	Diagnostics barriers and innovations in rural areas: insights from junior medical doctors on the frontlines of rural care in Peru	To explore the perspectives of medical doctors working in rural areas of Peru regarding the barriers impacting the diagnostic process, and ideas for diagnostic innovations that could assist them; of which a primary barrier is the lack of access to POC diagnostic tools
5	Araújo, W. J; Quirino, E. M. B.; Pinho, C. M.; Andrade, M. S.	2018	Revista Brasileira de Enfermagem	Perception of nurses who perform rapid tests in Health Centers	To understand the perception of health professionals who perform HIV rapid tests in Health Centres in Brazil, particularly the issues related to performing rapid tests and the opportunities for improvement in professional training and educational activities
6	Baltzell, K.; Elfving, K.; Shakely, D.; Ali, A. S.; Msellem, M.; Gulati, S.; Martensson, A.	2013	Malaria Journal	Febrile illness management in children under five years of age: a qualitative pilot study on primary health care workers' practices in Zanzibar	To explore health workers' practices and perspectives with febrile children under five years of age in Zanzibarian rural health facilities, and to identify factors influencing their diagnostic and management decisions in non-malarial fever patients

7	Bayer, A. M.; Najarro, L.; Zevallos, M.; Garcia, P. J.	2014	Reproductive Health	Potential point of care tests (POCTs) for maternal health in Peru, perspectives of pregnant women and their partners	To describe the demand side for pregnancy-related diagnostic tests from the perspective of Peruvian female and male community members, by engaging participants about their awareness of and care-seeking for pregnancy-related diagnostic tests (including anaemia, urinary infection and HIV) and their preferred characteristics and testing conditions for pregnancy-related POCTs
8	Beckham, S. W.; Karver, T. S.; Mantsios, A.; Shembilu, C.; Donastorg, Y.; Perez, M.; Gomez, H.; Barrington, C.; Mwampashi, A.; Davis, W.; Likindikoki, S.; Mbwambo, J. K.; Kerrigan, D.	2022	Global Public Health	Acceptability and perceptions of HIV oral self-testing across settings: a comparative qualitative study among Dominican and Tanzanian female sex workers	To explore perceived acceptability of rapid oral HIV self-tests (HIVST) among female sex workers in the Dominican Republic.
9	Beckmann, N.; Skovdal, M.; Maswera, R.; Nyamukapa, C.; Gregson, S.	2022	Global Public Health	Rituals of care: Strategies adopted by HIV testers to avoid misdiagnosis in rapid HIV testing in Zimbabwe	To explore how testers' ritualisation of laboratory practices and quality assurance procedures create a work environment conducive to high-quality testing using HIV rapid tests, via interviews with HIV testers and observations in four health facilities in Zimbabwe.
10	Beisel U., Umlauf R., Hutchinson E., Chandler C. I. R.	2016	Malaria Journal	The complexities of simple technologies: Re-imagining the role of rapid diagnostic tests in malaria control efforts	To explore the validity of the assumptions that malarial RDTs are simple to use and help to standardize diagnoses by examining their use in everyday settings in Uganda, Tanzania and Sierra Leone from the perspectives of formal and informal healthcare providers.
11	Bien, C. H.; Muessig, K. E.; Lee, R.; Lo, E. J.; Yang, LiGang; Yang, Bin; Peeling, R. W.; Tucker, J. D.	2015	PLoS ONE	HIV and syphilis testing preferences among men who have sex with men in South China: a qualitative analysis to inform sexual health services	To assess preferences for HIV and syphilis testing (HIV RDT, HIVST, syphilis RDT) among men who have sex with men in China in the context of facilitators and barriers to testing and of previous testing experiences.
12	Bjertrup, P. J.	2021	Medicine Anthropology Theory	(In)visible disease : motions and emotions engendered by papers and diagnostics of people accessing healthcare in Burkina Faso.	To explore how POCTs (malaria and dengue RDTs) shape people's diagnostic journeys within the health system in Burkina Faso; from the perspective of sick people and their families in the home, community and primary health care settings.
13	Boadu, N. Y. A.	2014	Thesis	Using Malaria Rapid Diagnostic Tests in Ghana: Understanding Healthcare Providers' Compliance with Policy Guidelines for Malaria Diagnosis in Peripheral Facilities	To investigate healthcare providers' perspectives of guideline compliance in direct relation to RDT use for malaria testing at their facilities.

14	Boadu, N. A.; Amuasi, J.; Ansong, D.; Einsiedel, E.; Menon, D.; Yanow, S. K.	2016	Malaria Journal	Challenges with implementing malaria rapid diagnostic tests at primary care facilities in a Ghanaian district: a qualitative study	To identify the factors directly influencing malaria RDT implementation at primary care facilities in Ghana from the perspective of providers.
15	Boakye, M. D. S.; Owek C. J.; Oluoch, E.; Wachira, J.; and Afrane, Y.A.	2018	BMC Public Health	Challenges of achieving sustainable community health services for community case management of malaria	To identify challenges of achieving sustainable community health services for Community Case Management of malaria in Kenya from the perspectives of community health workers, mothers of children under-five years, and public health officers and clinicians
16	Bones Rocha, K.; Grecco dos Santos, R. R.; Conz, J.; Tittoni da Silveira, A. C.	2016	Saúde em Debate	Transversalizando a rede: o matriciamento na descentralização do aconselhamento e teste rápido para HIV, sífilis e hepatites	To investigate the decentralization policy in the offering of rapid testing and counselling for HIV, syphilis and hepatitis RDTs at primary health services
17	Bwalya, C.; Simwinga, M.; Hensen, B.; Gwanu, L.; Hang'Andu, A.; Mulubwa, C.; Phiri, M.; Hayes, R.; Fidler, S.; Mwinga, A.; Ayles, H.; Ayles, H.; Bond, V.; Bond, V.	2020	AIDS Research and Therapy 2020	Social response to the delivery of HIV self-testing in households: Experiences from four Zambian HPTN 071 (PopART) urban communities	To explore the social response to and implications of door-to-door distribution of HIV self-testing kits, including secondary distribution of HIVST on household relations and the ability of individuals to self-test with or without supervision within households; in four Zambian communities, from the perspectives of Community HIV care providers and household members
18	Caixeta, E.R.; Coimbra, M. A. R.; Gomes, N. S.; Santana, L. C.; Delfino, F. A. P.; Ferreira, L. A.	2021	Rev Enferm UERJ	Percepção dos enfermeiros quanto ao acolhimento às pessoas que realizam o teste rápido de HIV	To analyse nurses' perceptions of receptiveness to people undergoing rapid HIV-testing
19	Carvalho, Fernanda T.; Both, Nalu S.; Alnoch, Edi M.; Conz, Jaqueline; Rocha, Katia B.	2016	Journal of Health Psychology	Counselling in STD/HIV/AIDS in the context of rapid test: Perception of users and health professionals at a counselling and testing centre in Porto Alegre	To discuss professionals' perceptions on counselling, users' perceptions on counselling and changes in counselling due to the introduction of HIV rapid test procedures at a counselling and testing centre in Brazil
20	Chandler, C. I. R.; Hall-Clifford, R.; Asaph, T.; Pascal, M.; Clarke, S.; Mbonye, A. K.	2011	Social Science and Medicine	Introducing malaria rapid diagnostic tests at registered drug shops in Uganda: Limitations of diagnostic testing in the reality of diagnosis	To explore how drug shops could be engaged in the diffusion of malaria RDTs in Uganda, from the perspective of drug shop workers, health workers, district health officials, and community members in community and primary health care settings
21	Chandler, C. I.; Whitty, C. J.; Ansah, E.	2010	American Journal of Tropical Medicine and Hygiene	How can malaria rapid diagnostic tests achieve their potential? A qualitative study of a trial at health facilities in Ghana	To explore the experiences of using malaria RDTs and their results amongst health workers, ranging from those who continued to prescribe anti-malarials to most patients with negative RDT results to those who largely restricted anti-malarials to patients with positive RDT results

22	Chipwaza, B.; Mugasa, J. P.; Mayumana, I.; Amuri, M.; Makungu, C.; Gwakisa, P. S.	2014	American Journal of Tropical Medicine and Hygiene	Community knowledge and attitudes and health workers' practices regarding non-malaria febrile illnesses in Eastern Tanzania	To assess the knowledge and attitude of communities regarding non-malaria febrile illnesses and health workers' practices related to diagnosis and treatment of febrile patients in Tanzania, from the perspectives of both community members and health workers
23	Choko, A. T.; Kumwenda, M. K.; Johnson, C. C.; Sakala, D. W.; Chikalipo, M. C.; Fielding, K.; Chikovore, J.; Desmond, N.; Corbett, E. L.	2017	Journal of the International AIDS Society	Acceptability of woman-delivered HIV self-testing to the male partner, and additional interventions: a qualitative study of antenatal care participants in Malawi	To understand the views of pregnant women and their male partners on HIV self-test kits that are woman-delivered, alone or with a linkage intervention, in Malawi
24	Cohen, J.; Cox, A.; Dickens, W.; Maloney, K.; Lam, F.; Fink, G.	2015	Malaria Journal	Determinants of malaria diagnostic uptake in the retail sector: qualitative analysis from focus groups in Uganda	To explore incentives and barriers to seeking diagnosis for malaria, how people react to test results and why, and what can be done to increase the willingness to pay for RDTs in Uganda, from the perspective of community members in the context of malaria RDTs' availability in private sector drug shops
25	Cross, J.; Street, A.	2022	Social Anthropology	To fail at scale! : minimalism and maximalism in humanitarian entrepreneurship	To trace the work involved in scaling-up the humanitarian effects of point-of-care diagnostic devices and solar-powered lanterns through processes of design, manufacturing and distribution from an anthropological perspective
26	da Silva, I. T. S.; Nogueira Valença, C.; da Silva, R. A. R.	2017	Anna Nery School Journal of Nursing	Mapping the implementation of the rapid HIV test in the Family Health Strategy: the nurses' perspective	To analyse the implementation of the rapid HIV test in Brazil's Family Health Strategy from the perspective of nurses
27	Dassah, E. T.; Adu-Sarkodie, Y.; Mayaud, P	2018	BMC Health Services Research	Rollout of rapid point of care tests for antenatal syphilis screening in Ghana: healthcare provider perspectives and experiences	To explore healthcare providers' experiences and challenges in antenatal syphilis screening following the national rollout of rapid syphilis POCTs in Ghana
28	Diggle, E.; Asgary, R.; Gore-Langton, G.; Nahashon, E.; Mungai, J.; Harrison, R.; Abagira, A.; Eves, K.; Grigoryan, Z.; Soti, D.; Juma, E.; Allan, R	2014	Malaria Journal	Perceptions of malaria and acceptance of rapid diagnostic tests and related treatment practises among community members and health care providers in Greater Garissa, North Eastern Province, Kenya	To identify and understand perceptions regarding malaria diagnosis and treatment, with a particular focus on RDTs, among community members and health care workers in Kenya
29	Engel, N.	2012	Biosocieties	New diagnostics for multi-drug resistant tuberculosis in India: Innovating control and controlling innovation	To examine the interplay between innovation of diagnostics for multi-drug resistant tuberculosis in India and control through standardization of operational and technical processes from a multidisciplinary perspective of actors engaged in diagnostic innovation activities at every level

30	Engel, N.; Ganesh, G.; Patil, M.; Yellappa, V.; Pai, N. P.; Vadnais, C.; Pai, M.	2015	PLoS ONE	Barriers to point-of-care testing in India: Results from qualitative research across different settings, users and major diseases	To identify potential barriers to the successful implementation of point-of-care testing programs in India across different points of care in health systems, considering the perspectives of formal and informal healthcare providers, patients, test manufacturers, laboratory technicians, program managers and policy-makers
31	Engel, N.; Davids, M.; Blankvoort, N.; Pai, Nitika P.; Dheda, K.; Pai, M.	2015	Tropical Medicine & International Health	Compounding diagnostic delays: a qualitative study of point-of-care testing in South Africa	To explore what POC tests are done and how in public/private, rural/urban hospitals and clinics in South Africa and whether they can ensure successful POC testing, considering the perspectives of doctors, nurses, community health workers, patients, laboratory technicians, policymakers, hospital managers and diagnostic manufacturers
32	Engel N.; Ganesh G.; Patil M.; Yellappa V.; Vadnais C.; Pai N.P.; Pai M.	2015	BMC Health Services Research	Point-of-care testing in India: Missed opportunities to realize the true potential of point-of-care testing programs	To identify potential barriers to the successful implementation of point-of-care testing programs in India across different points of care in health systems, considering the perspectives of formal and informal healthcare providers, patients, test manufacturers, laboratory technicians, program managers and policy-makers
33	Engel, N.; Yellappa, V.; Pai, N. P.; Pai, M.	2017	Science and Technology Studies	Diagnosing at Point of Care in South India: Coordination Work and Frictions	To examine the work involved in enacting point-of-care testing in India, and how technology and the embedded assumptions regarding patients feature in these enactments, considering the perspectives of community health workers, patients, laboratory technicians, tuberculosis programme staff and medical officers
34	Engel N.; Davids M.; Blankvoort N.; Dheda K.; Pant Pai N.; Pai M.	2017	BMC Health Services Research	Making HIV testing work at the point of care in South Africa: A qualitative study of diagnostic practices	To examine strategies of providers and clients in South Africa to make HIV testing successful in settings short of equipment, human resources and space, considering the perspectives of doctors, nurses, community health workers, patients, laboratory technicians, policymakers, hospital managers and manufacturers
35	Engel, N.; Wolffs, P. F. G.	2020	BMJ Global Health	Aligning diagnostics to the point-of-care: lessons for innovators, evaluators and decision-makers from tuberculosis and HIV	To explore how those engaged with developing and implementing new POC diagnostics for TB and HIV in India ensure these technologies work at POC, from the perspective of global stakeholders, providers, decision-makers, scientists and developers
36	Engel, N.	2020	Social Studies of Science	Aligning in the dark: Variable and shifting (user-) settings in developing point-of-care diagnostics for tuberculosis and HIV	To examine the innovation process of POC diagnostics for HIV and TB, and in particular the efforts to attune to local contexts and align multiple actors, settings and development steps.

37	Engel, N.	2020	In: Understanding Tuberculosis and its Control	Innovating Tuberculosis Diagnostics for the Point of Care	To trace attempts to innovate TB diagnostics from global to local level
38	Engel, N.; Krumeich, A.	2020	Frontiers in Sociology	Valuing Simplicity: Developing a Good Point of Care Diagnostic	To examine how norms and values of what constitutes a good point-of-care diagnostic are operating in practice in the context of tuberculosis and HIV testing, by considering the perspectives of diagnostic manufacturers, scientists, donors, members of civil society, industry consultants, international organizations, regulators, policymakers, programme officers, lab technicians, and clinicians
39	Epps, A.; Albury, C.; Van Hecke, O.	2021	Diagnostics	Exploring primary care clinicians' views about how best to implement a potential trial around point-of-care tests for common infections in south africa	To explore the barriers and facilitators for a future trial of POCTs for common infections in South Africa according to primary care clinicians
40	Evans, C.; Ndirangu, E	2011	Aids Care-Psychological and Socio-Medical Aspects of Aids/Hiv	Implementing routine provider-initiated HIV testing in public health care facilities in Kenya: a qualitative descriptive study of nurses' experiences	To investigate key challenges in provider-initiated testing and counselling for HIV from a nursing perspective, and to explore ways in which the principle of the 3Cs (consent, confidentiality and counselling) was being managed in everyday practice in Kenya
41	Febir, L. G.; Baiden, F. E.; Agula, J; Delimini, R. K.; Akpalu, B.; Tivura, M.; Amanfo, N.; Chandramohan, D.; Owusu-Agyei, S.; Webster, J.	2015	Malaria Journal	Implementation of the integrated management of childhood illness with parasitological diagnosis of malaria in rural Ghana: health worker perceptions	To explore perceptions in Ghana of health workers at various levels on the health system factors influencing effective delivery of test-based diagnosis of malaria with Integrated Management of Childhood Illness
42	Fonseca, P. de L; Iriart, J. A. B.	2012	Interface - Comunicação, Saúde, Educação	Aconselhamento em DST/Aids às gestantes que realizaram o teste anti-HIV na admissão para o parto: os sentidos de uma prática	To analyse the practices and meanings of counselling for pregnant women who underwent HIV testing on admission for deliver, and for healthcare professionals working in childbirth care
43	Guedes, H. C. dos S., Silva Júnior, J. N. de B., Henriques, A. H. B., Trigueiro, D. R. S. G., Nogueira, J. A., & Barrêto, A. J. R.	2021	Rev. Baiana Enferm	Discurso de gerentes sobre barreiras de acesso ao teste rápido anti-hiv na atenção primária	To study the barriers to access rapid HIV tests from the perspective of managers of primary healthcare services
44	Haenssgen, M. J.; Charoenboon, N.; Althaus, T.; Greer, R. C.; Intralawan, D.; Lubell, Y.	2018	Social Science & Medicine	The social role of C-reactive protein point-of-care testing to guide antibiotic prescription in Northern Thailand	To investigate the social role of C-reactive-protein point-of-care testing (CRP POCT) in Thailand through its interactions with (a) the healthcare workers who use it, (b) the patients whose routine care is affected by the test, and (c) the existing patient-health system linkages that might resonate or interfere with CRP POCT, according to

					the experiences of fever patients and their healthcare workers
45	Haenssgen, M. J.; Charoenboon, N.; Do, N. T. T.; Althaus, T.; Khine Zaw, Y.; Wertheim, H. F. L.; Lubell, Y.	2019	Trials	How context can impact clinical trials: a multi-country qualitative case study comparison of diagnostic biomarker test interventions	To investigate the introduction of a rapid diagnostic biomarker test (C-reactive protein, or CRP) to guide antibiotic prescriptions in outpatient settings, considering which factors account for cross-country variations in the effectiveness of CRP biomarker test interventions from the perspectives of healthcare workers and participants in Thailand, Myanmar, and Vietnam
46	Hardon, A.; Kageha, E.; Kinsman, J.; Kyaddondo, D.; Wanyenze, R.; Obermeyer, C. M.	2011	Medical Anthropology	Dynamics of Care, Situations of Choice: HIV Tests in Times of ART	To explore the perspectives of people attending testing services and of the nurses and counsellors conducting tests both in voluntary- and provider-initiated testing for HIV in Kenya and Uganda
47	Hayashi, K.; Lianping, Ti.; Kerr, T.; Hattirat, S.; Suwannawong, P.; Kaplan, K.; Ti, L.	2015	Sexual Health	Drug users stick together': HIV testing in peer-based drop-in centres among people who inject drugs in Thailand	To explore perspectives of people who inject drugs in Thailand on the acceptability of peer-based HIV testing through examination of past experiences, perceptions, interest, and potential concerns
48	Hill, J.; Landuwulang, C. U. R.; Ansariadi,; Hoyt, J.; Burdam, F. H.; Bonsapia, I.; Syafruddin, D.; Poespoprodjo, J. R.; Kuile, F. O. ter; Ahmed, R.; Webster, J.	2018	Malaria Journal	Evaluation of the national policy of single screening and treatment for the prevention of malaria in pregnancy in two districts in Eastern Indonesia: health provider perceptions	To assess health providers' acceptability and perceptions on the feasibility of implementing the single screening and treatment strategy for malaria in pregnancy in the context of the national programme in Indonesia
49	Hill, J.; Hoyt, J.; Achieng, F.; Ouma, P.; L'Lanziva, A.; Kariuki, S.; Desai, M.; Webster, J.	2016	PLoS ONE	User and Provider Acceptability of Intermittent Screening and Treatment and Intermittent Preventive Treatment with Dihydroartemisinin-Piperaquine to Prevent Malaria in Pregnancy in Western Kenya	To assess the acceptability among pregnant women and health providers of intermittent screening and treatment (ISTp) with sulphadoxine-pyrimethamine (SP) and intermittent preventive treatment in pregnancy (IPTp) with dihydroartemisinin-piperaquine (DP) as alternative strategies for reducing the risks associated with malaria in pregnancy in the context of an un-blinded clinical trial in Kenya
50	Hoyt, J.; Landuwulang, C. U. R.; Ansariadi,; Ahmed, R.; Burdam, F. H.; Bonsapia, I.; Poespoprodjo, J. R.; Syafruddin, D.; Kuile, F. O. ter; Webster, J.; Hill, J.	2018	Malaria Journal	Intermittent screening and treatment or intermittent preventive treatment compared to current policy of single screening and treatment for the prevention of malaria in pregnancy in	To assess the acceptability among health providers and pregnant women of the current 'single screen and treat' (SSTp) strategy compared to two alternative strategies in the context of a clinical trial in two malaria endemic provinces of Eastern Indonesia

				Eastern Indonesia: acceptability among health providers and pregnant women	
51	Hoyt, J.; Hill, J.; Achieng, F.; Ouma, P.; Kariuki, S.; Desai, M.; Webster, J.	2021	Malaria Journal	Healthcare provider and pregnant women's perspectives on the implementation of intermittent screening and treatment with dihydroartemisinin-piperaquine for malaria in pregnancy in western Kenya: a qualitative study	To evaluate the implementation feasibility of intermittent screening and treatment (ISTp) with dihydroartemisinin–piperaquine (DP) in routine antenatal care settings in Kenya, from the perspectives of both healthcare providers and pregnant women
52	Hutchinson, E.; Chandler, C.; Clarke, S.; Lal, S.; Magnussen, P.; Kayendeke, M.; Nabirye, C.; Kizito, J.; Mbonye, A	2015	Critical Public Health	It puts life in us and we feel big': shifts in the local health care system during the introduction of rapid diagnostic tests for malaria into drug shops in Uganda	To analyse the social interaction between drug sellers, their clients, and local health care workers regarding the introduction of rapid diagnostic tests for malaria into private sector drug shops in Uganda
53	Hutchinson, E.; Reyburn, H.; Hamlyn, E.; Long, K.; Meta, J.; Mbakilwa, H.; Chandler, C.	2017	Global Public Health	Bringing the state into the clinic? Incorporating the rapid diagnostic test for malaria into routine practice in Tanzanian primary healthcare facilities	To examine the introduction of the rapid diagnostic test for malaria (mRDT) into low-level public health facilities in Tanzania, considering the perspectives of health workers on how a participatory training programme, mobile phone messages, posters and leaflets shaped the use and interpretation of the test
54	Jain, K.; Mshweshwe-Pakela, N. T.; Charalambous, S.; Mabuto, Tonderai.; Hoffmann, C. J.	2019	AIDS Care	Enhancing value and lowering costs of care: a qualitative exploration of a randomized linkage to care intervention in South Africa	To explore values and costs of seeking clinical care for HIV in South Africa through testing three strategies to improve linkage to care: Point of care CD4 testing alone (POC-CD4), POC-CD4 combined with transportation support, and POC-CD4 combined with care facilitation; examined from the perspective of participants
55	Janssen, R.; Engel, N.; Esmail, A.; Oelofse, S.; Krumeich, A.; Dheda, K.; Pai, N. P.	2020	AIDS and Behavior	Alone But Supported: A Qualitative Study of an HIV Self-testing App in an Observational Cohort Study in South Africa	To investigate an oral HIV self-testing strategy together with a mobile phone/tablet application to see if and how it provided counselling and support, and how it might impact test access in South Africa from the perspective of both test users and trained test counsellors
56	Janssen, R.; Engel, N.; Pai, N. P.; Esmail, A.; Dheda, K.; Thomas, R.; Krumeich, A.	2021	Sociology of Health & Illness	You're only there on the phone'? A qualitative exploration of community, affect and agential capacity in HIV self-testing	To explore affective dimensions of HIV self-testing using a smartphone app strategy in South Africa and Canada from the perspectives of patients and trained HIV healthcare providers

				using a smartphone app	
57	Janssen, R.; Krumeich, A.; Esmail, A.; Thomas, R.; Dheda, K.; Pai, N.P.; and Engel, N.	2021	Medicine Anthropology Theory	Moments of Uncertainty Exploring How an App-Based Oral HIV Self-Testing Strategy Fits in Communities 'Living Under' HIV Risk	To explore how the introduction of an oral app-based HIVST strategy comes to work alongside existing HIV testing technologies and routines in South Africa from the perspectives of healthcare workers, nurses, doctors and test-users.
58	Jegede, A. S.; Oshiname, F. O.; Sanou, A. K.; Nsungwa-Sabiiti, J.; Ajayi, IO. O.; Siribie, M.; Afonne, C.; Serme, L.; Falade, C. O.	2016	Clinical Infectious Diseases	Assessing Acceptability of a Diagnostic and Malaria Treatment Package Delivered by Community Health Workers in Malaria-Endemic Settings of Burkina Faso, Nigeria, and Uganda	To assess acceptability of use of community health workers for case management of malaria using rapid diagnostic tests, artemisinin-based combination therapy, and rectal artesunate in Burkina Faso, Nigeria, and Uganda from the perspective of parents of sick children, community leaders, and health workers
59	Jennings, L.; Conserve, D. F.; Kajula, L.; Iwelunmor, J.; Maman, S.	2017	Annals of Global Health	Perceived cost advantages and disadvantages of purchasing HIV self-testing kits among urban tanzanian men: An inductive content analysis	To assess perceived costs saved and costs incurred from use of HIV self-testing kits in infrequently- or never-tested Tanzanian men
60	Johansson, E. W.; Kitutu, F. E.; Mayora, C.; Awor, P.; Peterson, S. S.; Wamani, H.; Hildenwall, H.	2016	Malaria Journal	It could be viral but you don't know, you have not diagnosed it: health worker challenges in managing non-malaria paediatric fevers in the low transmission area of Mbarara District, Uganda	To explore perceptions of rapid diagnostic testing for malaria in children in Uganda among health workers and caregivers, as well as the factors and influences on health workers' management of nonmalaria febrile paediatric patients, and caregivers' experiences of care for nonmalaria fevers
61	Kabwama, S. N.; Kiwanuka, S. N.; Mapatano, M. A.; Fawole, O. I.; Seck, I.; Namale, A.; Ndejjo, R.; Kizito, S.; Monje, F.; Bosonkie, M.; Egbende, L.; Bello, S.; Bamgboye, E. A.; Dairo, M. D.; Adebowale, A. S.; Salawu, M. M.; Afolabi, R. F.; Diallo, I; Leye, M. M. M.; Ndiaye, Y.	2022	Globalization & Health	Private sector engagement in the COVID-19 response: experiences and lessons from the Democratic Republic of Congo, Nigeria, Senegal and Uganda	To assess responses to the COVID-19 pandemic and the public and private sector partnerships and engagements which emerged to address critical gaps in the response, specifically in Democratic Republic of Congo, Nigeria, Senegal and Uganda between November 2020 and March 2021 from the perspectives of private and public health sector stakeholders
62	Kameda, K.; Kelly, A. H.; Lezaun, J.; Löwy, I.	2021	Social Studies of Science	Imperfect diagnosis: The truncated legacies of Zika testing	To analyse the limited impact of global R&D efforts on the availability of Zika diagnostic options, and the ways testing during the Zika crisis reveal some of the fault lines in the global health enterprise from the perspectives

					of key actors involved in diagnostic R&D efforts during the Zika emergency
63	Katirayi, L.; Ochuka, B.; Mafaune, H.; Chadambuka, A.; Baffour, T.; Sacks, E.	2020	Journal of Acquired Immune Deficiency Syndromes	"We Need it the Same Day": A Qualitative Study of Caregivers and Community Members' Perspectives Toward the Use of Point-of-Care Early Infant Diagnosis	To understand the acceptability of POC among caregivers of HIV-exposed infants and community members in Kenya and Zimbabwe
64	Kelly, A. H.; Lezaun, J.; Street, A.	2022	Economy and Society	Global health, accelerated: Rapid diagnostics and the fragile solidarities of 'emergency R&D'	To examine the contours of an emerging regime of emergency research and development in global health and how its related epistemic practices and ethical norms played out and shifted in the development of rapid diagnostic tests for Ebola, Zika and COVID-19
65	Khine Zaw, Y.; Charoenboon, N.; Haenssgen, M. J.; Lubell, Y.	2018	The American Journal of Tropical Medicine and Hygiene	A Comparison of Patients' Local Conceptions of Illness and Medicines in the Context of C-Reactive Protein Biomarker Testing in Chiang Rai and Yangon	To compare local conceptions of illness and medicines in relation to health-care seeking and antibiotic demand, and to understand how these conceptions could influence CRP point-of-care testing, from the perspectives of febrile patients in Thailand and Myanmar
66	Kitchen, P. J.; Barnighausen, K.; Dube, L.; Mnisi, Z.; Dlamini-Nqeketo, S.; Johnson, C. C.; Barnighausen, T.; De Neve, J. W.; McMahon, S. A.	2020	African Journal of AIDS Research	Expansion of HIV testing in Eswatini: stakeholder perspectives on reaching the first 90	To elicit the facilitators and inhibitors to HIV testing uptake in Eswatini from the perspective of stakeholders from policy, implementation, donor, local advocacy and academic sectors
67	Knight, L. C.; Rooyen, H. van; Humphries, H.; Barnabas, R. V.; Celum, C.	2015	AIDS Care	Empowering patients to link to care and treatment: qualitative findings about the role of a home-based HIV counselling, testing and linkage intervention in South Africa	To explore the barriers and facilitators of linkage to and retention in care amongst persons who tested positive for HIV in rural South Africa, considering the perspectives of HIV-positive patients, intervention research counsellors, and government clinic staff
68	Kroeger, K.; Taylor, A.; Marlow, H.; Fleming, D. T.; Beyleveld, V.; Alwano, M. G.; Kejelepula, M. T.; Chilume, K. B.; Smith, D. K.; Roels, T. H.; Kilmarx, P. H.& Peter Kilmarx	2011	SAHARA-J: Journal of Social Aspects of HIV/AIDS	Perceptions of door-to-door HIV counselling and testing in Botswana	To assess and document community member's perceptions about the concept of door-to-door HIV counselling and rapid testing in two of the highest-prevalence districts of Botswana
69	Lee, R.	2015	Havard dissertations	Decentralized STD Testing Among Men Who Have Sex with Men in South China -	To assessed preferences in China for HIV and syphilis testing among men who have sex with men in the context

				A Qualitative Analysis to Inform Sexual Health Services	of facilitators and barriers to testing and previous testing experiences
70	Lee, S. J.; Palmer, J. J.	2018	Infectious Diseases of Poverty	Integrating innovations: a qualitative analysis of referral non-completion among rapid diagnostic test-positive patients in Uganda's human African trypanosomiasis elimination programme	To explore why some human African trypanosomiasis-positive patients who receive diagnosis from a rapid diagnostic test do not complete the diagnostic referral process in Uganda, and to examine their experiences of each step of the referral process
71	Ley, B.; Thriemer, K.; Jaswal, J.; Poirot, E.; Alam, M. S.; Phru, ChingSwe; Khan, W. A.; Dysoley, L.; Qi, Gao; Kheong, ChongChee; Shamsudin, U. K.; Chen, I.; Hwang, JiMee; Gosling, R.; Price, R. N.	2017	Malaria Journal	Barriers to routine G6PD testing prior to treatment with primaquine	To examine policy makers' and healthcare providers' perceptions of vivax malaria, -primaquine based treatment for malaria and the complexities of glucose-6-phosphate-dehydrogenase deficiency, which can increase the risk of drug-induced haemolysis, in Bangladesh, Cambodia, China, and Malaysia
72	Lofgren, S. M.; Nalintya, E; Meya, D. B.; Boulware, D. R.; Rajasingham, R.	2018	Medicine	A qualitative evaluation of an implementation study for cryptococcal antigen screening and treatment in Uganda	To identify barriers to implementation as well as facilitating factors for cryptococcal antigen screening programs' success from the perspective of health care workers in Uganda
73	Lohfeld, L.; Kangombe-Ngwenya, T.; Winters, A. M.; Chisha, Z.; Hamainza, B.; Kamuliwo, M.; Miller, J. M.; Burns, M.; Bridges, D. J.	2016	Malaria Journal	A qualitative review of implementer perceptions of the national community-level malaria surveillance system in Southern Province, Zambia	To determine perceptions around malaria rates, incentives, operational challenges and solutions according to CHWs, their supervisors and district-level managers related to reactive case detection of malaria at a community level in rural Zambia
74	Macharia, L. W.; Wexler, C.; Brown, M.; Maloba, M.; Pricilla, R. A.; Mabachi, N. M.; Muchoki, E.; Babu, S.; Ochieng, M.; Gautney, B.; Goggin, K.; Finocchario-Kessler, S.	2020	PLoS ONE	Implementation planning for community-based point-of-care HIV testing for infants: Recommendations from community leaders in Kenya	To explore the acceptability and feasibility of community-based POC HIV testing from the perspective of community health workers and volunteers, traditional birth attendants, community leaders, and parents of HIV-exposed infants in Kenya
75	Maddox, B. L. P.; Wright, Shaunta S.; Namadingo, H.; Bowen, V. B.; Chipungu, G. A.; Kamb, M. L.	2017	Sexually Transmitted Infections	Assessing stakeholder perceptions of the acceptability and feasibility of national scale-up for a dual HIV/syphilis rapid diagnostic test in Malawi	To explore the importance of a dual rapid diagnostic test for HIV and syphilis among pregnant women, concerns using and procuring the dual test, and recommendations for national expansion from the perspective of healthcare workers, laboratorians,

					Ministry of Health leaders and partner agency representatives in Malawi
76	Marks, M.; Esau, T.; Asugeni, R.; Harrington, R.; Diau, J.; Toloka, H.; Asugeni, J.; Ansbro, E.; Solomon, A. W.; Maclaren, D.; Redman-Maclaren, M.; Mabey, D. C. W.	2018	PLoS Neglected Tropical Diseases	Point-of-care tests for syphilis and yaws in a low-income setting - A qualitative study of healthcare worker and patient experiences	To evaluate healthcare worker and patient perceptions of using a syphilis/yaws POCT in clinics in the Solomon Islands
77	Martinez-Perez, G. Z.; Nikitin, D. S.; Bessonova, A.; Fajardo, E.; Bessonov, S.; Shilton, S.	2021	BMC Infectious Diseases	Values and preferences for hepatitis C self-testing among people who inject drugs in Kyrgyzstan	To assess values and preferences relating to hepatitis C virus self-testing among people who inject drugs in Kyrgyzstan; specifically, awareness of HCV and current HCV testing experiences, and acceptability and service delivery preferences for HCV self-testing
78	Mashamba-Thompson, T. P.; Jama, N. A.; Sartorius, B.; Drain, P. K.; Thompson, R. M	2017	Diagnostics	Implementation of point-of-care diagnostics in rural primary healthcare clinics in south africa: Perspectives of key stakeholders	To explore key stakeholder perceptions on the implementation of POC diagnostics in rural primary healthcare clinics in South Africa against the World Health Organisation (WHO) quality-ASSURED criteria
79	Mbonye, A. K.; Ndyomugyenyi, R.; Turinde, A.; Magnussen, P.; Clarke, S.; Chandler, C.	2010	Malaria Journal	The feasibility of introducing rapid diagnostic tests for malaria in drug shops in Uganda	To understand use of drug shops by communities in Uganda, particularly as potential outlets for provision of malaria RDTs, from the perspective of drug shop attendants, health workers and district health officials
80	McDowell, A.; Raizada, N.; Khaparde, S. D.; Rao, R.; Sarin, S.; Kalra, A.; Salhotra, V. S.; Nair, S. A.; Boehme, C.; Denkinger, C. M.	2018	PLoS ONE	"Before Xpert I only had my expertise": A qualitative study on the utilization and effects of Xpert technology among pediatricians in 4 Indian cities	To understand how national guidelines on TB diagnosis and Xpert technology have been integrated into the paediatric TB care practices of different health providers, considering perspectives of health providers from public and private sectors engaged in the ongoing paediatric project in 4 major cities of India
81	Mohamed, Y.; Kupul, M.; Gare, J.; Badman, S. G.; Silim, S.; Vallely, A. J.; Luchters, S.; Kelly-Hanku, A.	2020	BMJ Open	Feasibility and acceptability of implementing early infant diagnosis of HIV in Papua New Guinea at the point of care: A qualitative exploration of health worker and key informant perspectives	To explore the feasibility and acceptability of implementing a point-of-care (POC) EID test (Xpert HIV-1 Qualitative assay) among health workers and key stakeholders working within the prevention of mother-to-child transmission of HIV programme in Papua New Guinea

82	Mphwatiwa, T.; Witek-McManus, S.; Mtali, A.; Okello, G.; Nguluwe, P.; Chatsika, H.; Roschnik, N.; Halliday, K. E.; Brooker, S. J.; Mathanga, D.	2017	Malaria Journal	School-based diagnosis and treatment of malaria by teachers using rapid diagnostic tests and artemisinin-based combination therapy: experiences and perceptions of users and implementers of the Learner Treatment Kit, southern Malawi	To explore experiences and perceptions of a programme of school-based malaria case management via a first-aid kit in Malawi, from the perspectives of school children, parents and guardians, and teachers as well as key stakeholders at the school, district and national levels
83	Msimango, L.; Gibbs, A.; Shozi, H.; Ngobese, H.; Humphries, H.; Drain, P. K.; Garrett, N.; Dorward, J.	2020	BMC Health Services Research	Acceptability of point-of-care viral load testing to facilitate differentiated care: a qualitative assessment of people living with HIV and nurses in South Africa	To assess the acceptability of POC viral load testing within a differentiated care model that involved task-shifting from professional nurses to less highly trained enrolled nurses, and an option of collecting treatment from a community-based antiretroviral therapy delivery programme, from the perspectives of clients, professional and enrolled nurses in South Africa
84	Mukanga, D.; Tibenderana, J. K.; Kiguli, J.; Pariyo, G. W.; Waiswa, P.; Bajunirwe, F.; Mutamba, B.; Counihan, H.; Ojiambo, G.; Kallander, K.	2010	Malaria Journal	Community acceptability of use of rapid diagnostic tests for malaria by community health workers in Uganda	To assess community acceptability of the use of rapid diagnostic tests by Ugandan community health workers; to explore<br>issues of trust and confidence in CHWs, stigma associated with drawing blood from children, community willingness for CHWs to use RDTs, and challenges anticipated to be faced by the CHWs considering the perspectives of CHWs and caregivers of children under five years as well as health workers and community leaders
85	Mwangala, S.; Moland, K. M.; Nkamba, H. C.; Musonda, K. G.; Monze, M.; Musukwa, K. K.; Fylkesnes, K.	2015	PLoS ONE	Task-Shifting and Quality of HIV Testing Services: Experiences from a National Reference Hospital in Zambia	To explore various cadres of providers' experiences, including lay counsellors, nurses and laboratory personnel, in providing HIV testing services and their understanding of elements that impact on quality of service in Zambia
86	Mwaura, M.; Engel, N.	2021	International Society for Infectious Diseases	Constructing confidence: User perspectives on AlereLAM testing for tuberculosis	To understand perspectives and experiences of those using AlereLAM, a point-of-care test intended to address the challenges of diagnosing tuberculosis in people living with HIV, from the perspective of clinicians, nurses, program officers, laboratory staff, and patient advocates in Uganda, Kenya, and South Africa
87	Naidoo, P.; Niekerk, M. van; Toit, E. du; Beyers, N.; Leon, N.	2015	BMC Health Services Research	Pathways to multidrug-resistant tuberculosis diagnosis and treatment initiation: a qualitative comparison of patients' experiences in the era of rapid molecular diagnostic tests	To explore and compare multidrug-resistant tuberculosis patients' experiences of their diagnostic and treatment initiation pathway in the context of new rapid diagnostic tests, and to better understand to symptom recognition, health-care access, testing for MDR-TB and treatment initiation in South Africa

88	Ncube, V.; Chataway, J.	2019	Innovation and Development	Harnessing innovative HIV point-of-care testing for health systems strengthening: early lessons from Zimbabwe	to trace and analyse the deployment and uptake of innovative point-of-care testing in the early infant diagnosis of HIV in Zimbabwe from the perspective of policymakers
89	Ndakidemi, E.; Emerson, C.; Medley, A.; Ngowi, B.; Ng'eno, B.; Munuo, G.; Kohi, W.; Modi, S.	2019	International Union against Tuberculosis and Lung Disease	Health care worker perspectives on TB case finding and HIV services among pediatric TB patients in Tanzania	To identify the barriers to paediatric TB diagnosis and HIV care in Tanzania from the perspective of community health workers
90	Njau, B.; Ostermann, J.; Brown, D.; Muhlbacher, A.; Reddy, E.; Thielman, N.	2014	BMC Public Health	HIV testing preferences in Tanzania: a qualitative exploration of the importance of confidentiality, accessibility, and quality of service	To develop a conceptual framework for understanding which characteristics of HIV testing are associated with preferences for testing, and identify factors that influence whether and where people test for HIV in Tanzania from the perspective of community members
91	Nsagha, D. S.; Elat, J. B. N.; Ndong, P. A. B.; Tata, P. N.; Tayong, M. N. N.; Pokem, F. F.; Wankah, C. C.	2011	Drug, Healthcare and Patient Safety	Feasibility of home management using ACT for childhood malaria episodes in an urban setting	To determine community perception and the treatment response to episodes of childhood malaria in an urban setting prior to implementation of home management using artemisinin-based combination therapy by gathering perspectives of caregivers of children under 5 years in Cameroon
92	Nygren, D.; Isaksson, A. L.	2014	Journal of Public Health in Africa	Battling malaria in rural Zambia with modern technology: A qualitative study on the value of cell phones, geographical information systems, asymptomatic carriers and rapid diagnostic tests to identify, treat and control malaria	To evaluate the impact of cell phone surveillance and geographical information systems on malaria control in Zambia, and evaluate what screening and treatment of asymptomatic carriers and implementation of rapid diagnostic tests in rural health care has led to from the perspective of staff at different levels at the Macha Research Trust and at surrounding rural health centres
93	O'Neill, S.; Dierickx, S.; Okebe, J.; Dabira, E.; Gryseels, C.; D'Alessandro, U.; Grietens, K. P.	2016	PLoS ONE	The importance of blood is infinite: Conceptions of blood as life force, rumours and fear of trial participation in a Fulani village in rural Gambia	To explore anxieties around blood-taking during a malaria treatment trial in the Gambia, from the perspective of trial participants
94	O'Sullivan, M.; Kenilorea, G.; Yamaguchi, Y.; Bobogare, A.; Losi, L.; Atkinson, J. A.; Vallely, A.; Whittaker, M.; Tanner, M.; Wijesinghe, R.	2011	Malaria Journal	Malaria elimination in Isabel Province, Solomon Islands: Establishing a surveillance-response system to prevent introduction and reintroduction of malaria	To provide information on the feasibility and acceptability of implementing a new approach of surveillance and response that involves testing all travellers entering Isabel Province using rapid diagnostic tests (RDT), in the context of low levels of indigenous malaria transmission in Solomon Islands, from the perspectives of community members, local leaders, government stakeholders and healthcare workers

95	Ochoa-Manjarrés, M. T.; Gaitan-Duarte, H. G.; Caicedo, S.; Perez, B.G. y F.	2016	Rev Panam Salud Publica	Introducción de pruebas rápidas para sífilis y VIH en el control prenatal en Colombia: análisis cualitativo	Interpreting the perception of Colombian HCP about the barriers and facilitators for the introduction of rapid tests for syphilis and HIV in prenatal care services
96	Okoboi 2019	2019	Journal of the International AIDS Society	Acceptability, perceived reliability and challenges associated with distributing HIV self-test kits to young MSM in Uganda: a qualitative study	To assess the acceptability, perceived reliability and challenges associated with distributing HIV self-test to young men who have sex with men (MSM) in Uganda, from the perspectives of both MSM and health workers
97	Okoboi, Stephen; Twimukye, Adelline; Lazarus, Oucul; Castelnuovo, Barbara; Agaba, Collins; Immaculate, Muloni; Nanfuka, Mastula; Kambugu, Andrew; King, Rachel	2020	BMC Health Services Research	Improving the effectiveness of point of care tests for malaria and anaemia: a qualitative study across three Ghanaian antenatal clinics	To identify the current practice of POCT use for malaria and anaemia, to explore the enablers and barriers to effective implementation of these POCT, and to determine how relationships between each of the stakeholder groups may impact on POCT use in Ghana, from the perspectives of both clinical and laboratory staff as well as pregnant female patients
98	Palmer, T.; Aiyenigba, A. O.; Bates, I.; Okyere, D. D.; Tagbor, H.; Ampofo, G. D.	2019	PLoS Neglected Tropical Diseases	Potential Use of Community-Based Rapid Diagnostic Tests for Febrile Illnesses: Formative Research in Peru and Cambodia	To assess acceptability of use of rapid diagnostic tests for emerging infectious diseases including dengue, malaria, plague, and melioidosis in Peru and Cambodia from the perspectives of community members and health professionals
99	Paz-Soldan, V. A.; Morrison, A. C.; Sopheab, H.; Schwarz, J.; Bauer, K. M.; McKenney, J. L.; Chhea, C.; Saphonn, V.; Khuon, D.; Hontz, R. D.; Gorbach, P. M.	2014	Malaria Journal	The acceptability of intermittent screening and treatment versus intermittent preventive treatment during pregnancy: results from a qualitative study in Northern Ghana	To explore the acceptability of intermittent screening and treating, including use of mRDTs, compared to intermittent preventive treatment in the context of a clinical trial in Ghana, from the perspectives of pregnant female trial participants as well as clinical trial staff
100	Pell, C.; Menaca, A.; Chatio, S.; Hodgson, A.; Tagbor, H.; Pool, Robert.	2020	Rev. Enferm. UFSM - REUFSM	Realização de testes rápidos de sífilis em gestantes por enfermeiros da atenção básica	To understand how nurses perform rapid syphilis testing at pregnant women at primary healthcare services
101	Pereira, B. B.; dos Santos, C. P.; Gomes, G. C.	2018	Rev. Salud Pública	Strengths and weaknesses of rapid Hiv test implementation in a specialized center in Brazil	To analyse how health professionals and health service users in Brazil assess counselling on Rapid HIV test, as well as its implementation, focusing on positive aspects, barriers, and limitations, and analysing possible meanings that are given to STD and HIV/AIDS
102	Pizzinato, A; Machado, R. de O.; Carvalho, F. T. de.; Freire, I. N.; Bones Rocha, K	2022	AIDS	How informal healthcare providers improve uptake of HIV testing: qualitative results from a randomized controlled trial	To evaluate the acceptability and implementation of an HIV testing program where traditional healers delivered point-of-care testing and counselling to adults of unknown serostatus, from the perspective of both healers and community members

103	Ponticiello, M.; Mwanga-Amumpaire, J.; Tushemereirwe, P.; Nuwagaba, G.; Nansera, D.; King, R.; Muyindike, W.; Sundararajan, R.	2021	Aids Care-Psychological and Socio-Medical Aspects of Aids/Hiv	Adolescents' and caregivers' perceptions of caregiver-provided testing and HIV self-testing using oral mucosal transudate tests in Zimbabwe: a short report	To explore adolescents' perceptions of HIV self-testing (HIVST) and caregivers' perceptions of testing their children using an oral mucosal transudate (OMT) rapid HIV test in Zimbabwe
104	Rainer, C.; Chihota, B.; Chikwari, C. D.; McHugh, G.; Dauya, E.; Mujuru, H.; Ferrand, R. A.; Stewart, K. A.	2018	Tropical Medicine and Health	Antimalarial drug prescribing by healthcare workers when malaria testing is negative: A qualitative study in Madagascar	To explore why and how primary healthcare workers in Madagascar continue to prescribe antimalarial drugs to patients with negative results on malaria rapid diagnostic tests from the perspective of doctors and nurses
105	Rakotonandrasana, D. H.; Tsukahara, T.; Yamamoto-Mitani, N.	2017	PLoS ONE	Health care workers' perceptions of point-of-care testing in a low-income country-A qualitative study in Southwestern Uganda	To explore perceptions of POC test end-users in Uganda, including the use and characteristics of nineteen current tests, from the perspective of midwives, laboratory technicians, clinical and medical officers, junior and senior nurses, and medical doctors
106	Rasti, R.; Nanjebe, D.; Karlstrom, J.; Muchunguzi, C.; Mwanga-Amumpaire, J.; Gantelius, J.; Martensson, A.; Rivas, L.; Galban, F.; Reutersward, P.; Andersson Svahn, H.; Alvesson, H. M.; Boum II, Y.; Alfven, T.	2017	PeerJ	Implementation of the Xpert MTB/RIF assay for tuberculosis in Mongolia: A qualitative exploration of barriers and enablers	To identify barriers and enablers to implementation of the Xpert MTB/RIF test within Mongolia's National Tuberculosis Program from the perspectives of laboratory staff and tuberculosis physicians
107	Rendell, N. L.; Bekhbat, S.; Ganbaatar, G.; Dorjravdan, M.; Pai, M.; Dobler, C. C.	2013	Malaria Research and Treatment	Expanding access to malaria diagnosis through retail shops in western kenya: What do shop workers think?	To understand the perceptions of the benefits and challenges to selling rapid diagnostic tests for malaria from the perspective of antimalarial retailers in Kenya
108	Rusk, A.; Goodman, C.; Naanyu, V.; Koech, B.; Obala, A.; O'Meara, W. P.	2022	BMC Pediatrics	The baby will have the right beginning': a qualitative study on mother and health worker views on point-of-care HIV birth testing across 10 sites in Zimbabwe	To understand the views of mothers and health workers regarding the use and acceptability of POC birth testing for HIV in Zimbabwe
109	Sacks, E.; Katirayi, L.; Kaeberle, B.; Mafaune, H. W.; Chadambuka, A.; Tachiwenyika, E.; Nyamundaya, T.; Cohn, J.; Mahomva, A.; Mushavi, A.	2019	BMC Health Services Research	Qualitative assessment of South African healthcare worker perspectives on an instrument-free rapid CD4 test	To explore the views of healthcare workers in a large women and children's hospital on the acceptability and feasibility of an instrument-free point-of-care CD4 test for people living with HIV in South Africa

110	Scorgie, F.; Mohamed, Y.; Anderson, D.; Crowe, S. M.; Luchters, S.; Chersich, M. F.	2017	Journal of the International AIDS Society	Understanding uptake of an intervention to accelerate antiretroviral therapy initiation in Uganda via qualitative inquiry	To understand perceptions, attitudes and the context of changes in Antiretroviral Therapy (ART) initiation practices in Uganda, and specifically the rapid rise in ART initiation and provider behaviour change after the SMART-ART study, from the perspectives of healthcare providers and study staff
111	Semitala, F. C.; Camlin, C. S.; Wallenta, J.; Kampiire, L.; Katuramu, R; Amanyire, G; Namusobya, J.; Chang, W.; Kahn, J. G.; Charlebois, E. D.; Havlir, D. V.; Kamya, M. R.; Geng, E. H.	2016	Malaria Journal	Community perceptions of mass screening and treatment for malaria in Siaya County, western Kenya	To explore community perceptions of the intermittent mass screening and treatment intervention in Kenya, and specifically of testing and treatment in the absence of symptoms, before and after implementation of the intervention, from the perspectives of both community members and community health workers
112	Shuford, K.; Were, F.; Awino, N.; Samuels, A.; Ouma, P.; Kariuki, S.; Desai, M.; Allen, D. R.	2012	BMC Health Services Research	An assessment of the Zimbabwe ministry of health and child welfare provider initiated HIV testing and counselling programme	To evaluate institutional capacity to implement Provider-initiated HIV testing and counselling (PITC) and investigate patient and health care worker (HCW) perceptions of the PITC programme in Zimbabwe
113	Sibanda, E. L.; Hatzold, K.; Mugurungi, O.; Ncube, G.; Dupwa, B.; Siraha, P.; Madyira, L. K.; Mangwiro, A.; Bhattacharya, G.; Cowan, F. M.	2015	Malaria Journal	A qualitative study of perceptions of a mass test and treat campaign in Southern Zambia and potential barriers to effectiveness	To understand CHW and community perceptions regarding the malaria mass test and treat campaign (MTAT) using rapid diagnostic tests in Zambia
114	Silumbe, K.; Chiyende, E.; Finn, T. P.; Desmond, M.; Puta, C.; Hamainza, B.; Kamuliwo, M.; Larsen, D. A.; Eisele, T. P.; Miller, J.; Bennett, A.	2022	Medical Anthropology	The (In)visibility of Misdiagnosis in Point-of-Care HIV Testing in Zimbabwe	To explore how healthcare workers in Zimbabwe conducting rapid HIV tests ascertain the accuracy of their test results in contexts of limited external quality assurance mechanisms
115	Skovdal, M.; Beckmann, N.; Maswera, R.; Nyamukapa, C.; Gregson, S.	2020	PLoS ONE	Uncertainties, work conditions and testing biases: Potential pathways to misdiagnosis in point-of-care rapid HIV testing in Zimbabwe	To investigate potential pathways to misdiagnosis within point-of-care rapid HIV testing programmes in Zimbabwe from the perspectives of HIV testers
116	Skovdal, M.; Beckmann, N.; Maswera, R.; Nyamukapa, C.; Gregson, S.	2010	Malaria Journal	Intermittent screening and treatment versus intermittent preventive treatment of malaria in pregnancy: user acceptability	To compare the relative acceptability of intermittent preventive treatment (IPT) of malaria during pregnancy and intermittent screening and treatment (IST), which involves a rapid diagnostic test for malaria, in Ghana from the perspective of pregnant women enrolled in a trial comparing the efficacy of these two strategies

117	Smith, L. A.; Jones, C.; Adjei, R. O.; Antwi, G. D.; Afrah, N. A.; Greenwood, B.; Chandramohan, D.; Tagbor, H.; Webster, J.	2015	Malaria Journal	Motivation and challenges for use of malaria rapid diagnostic tests among informal providers in Myanmar: a qualitative study	To understand the acceptability of using RDTs in the informal sector in Myanmar, to examine motivations for use among informal providers and, to highlight decision-making and knowledge of providers for diagnostic testing and treatment, from the perspective of informal providers
118	Sudhinaraset, M.; Briegleb, C.; Aung, M.; Khin, H. S. S.; Aung, T.	2019	BMJ Open	Exploring global and country-level barriers to an effective supply of leishmaniasis medicines and diagnostics in eastern Africa: a qualitative study	To understand stakeholders' perceptions of the access barriers and identify key bottlenecks to quality-assured diagnostics and medicines for leishmaniasis from the perspectives of representatives from international organisations, non-governmental agencies, national control programmes from six countries (Ethiopia, Kenya, Somalia, South Sudan, Sudan and Uganda) and manufacturers
119	Sunyoto, T.; Potet, J.; den Boer, M.; Ritmeijer, K.; Postigo, J. A. R.; Ravinetto, R.; Alves, F.; Picado, A.; Boelaert, M.	2011	BMC Public Health	A qualitative exploration of the human resource policy implications of voluntary counselling and testing scale-up in Kenya: applying a model for policy analysis	To examine the human resource policy implications of scaling up HIV testing and counselling in Kenya and to analyse the resultant policy against a recognised theoretical framework of health policy reform from the perspectives of policy makers
120	Taegtmeyer, M.; Martineau, T.; Namwebya, J. H.; Ikahu, A.; Ngare, C. W.; Sakwa, J.; Lalloo, D. G.; Theobald, S.	2007	Rev Saúde Pública	Impressões sobre o teste rápido para o HIV entre usuários de drogas injetáveis no Brasil	To describe perceptions, experiences, knowledge, beliefs of injecting drug users around HIV RDTs and their willingness to be tested with such devices
121	Telles-Dias, P. R.; Westman, S.; Fernandez, A. E.; Sanchez, M.	2022	Global Health-Science and Practice	"Testing Can Be Done Anywhere": A Qualitative Assessment of Targeted Community-Based Point-of-Care Early Infant Diagnosis of HIV in Lusaka, Zambia	To explore the acceptability, appropriateness, and feasibility of deploying a targeted community-based point-of-care early infant diagnosis testing model to reach mother-infant pairs at high risk for HIV in Zambia by gathering the perspectives of health care workers, study staff, and high-risk mothers
122	Tembo, T.; Dale, H.; Muttau, N.; Itoh, M.; Williamson, D.; Mwamba, C.; Manasyan, A.; Beard, R. S.; Cox, M. H.; Herce, M. E.	2021	AIDS and Behavior	Point-of-Care Test for Assessing Tenofovir Adherence: Feasibility and Recommendations from Women in an Oral PrEP Program in Kenya and Their Healthcare Providers	To explore oral pre-exposure prophylaxis experiences and assess both feasibility and acceptability of a point-of-care urine test identifying recent non-adherence to tenofovir-based PrEP among female PrEP users and health care providers in Kenya
123	Thuo, N.; Polay, M.; Leddy, A. M.; Ngure, K.; Chatterhee, P.; Gandhi, M.; Amico, K. R.	2013	Malaria Journal	Qualitative study of presumptive treatment of childhood malaria in third tier tertiary hospitals in southeast Nigeria: a focus group and in-depth study	to determine patient, provider and laboratory attributes that sustain presumptive treatment of childhood-malaria in Nigeria, considering perspectives from parents/guardians, providers, and laboratory scientists

124	Ughasoro, M. D.; Okoli, C. C.; Uzochukwu, B. Sc.	2017	Medical Anthropology	Precarity and Preparedness: Non-Adherence as Institutional Work in Diagnosing and Treating Malaria in Uganda	To explore how novel regulation technologies like rapid diagnostic tests affect existing institutions of care, and how presumptive treatment includes a variety of practices, performances, temporalities, and opportunities that allow individuals to prepare for future episodes of fever from an academic anthropological perspective, specifically in a Ugandan context
125	Umlauf, R.	2018	Global Public Health	Stock-outs! Improvisations and processes of infrastructuring in Uganda's HIV/Aids and malaria programmes	To trace and compare interruptions in the supply of antiretrovirals and Rapid Diagnostic Tests in Uganda in order to provide an ethnographic account of the complex role that improvisations play within global health infrastructures
126	Umlauf, R.; Park, S-J.	2019	BMJ Open	Introducing new point-of-care tests for common infections in publicly funded clinics in South Africa: a qualitative study with primary care clinicians	To explore the perceptions of South African primary care clinicians working in publicly funded clinics about: making antibiotic prescribing decisions for two common infection syndromes (acute cough, urinary tract infection); their experiences of existing POCTs; their perceptions of the barriers and opportunities for introducing (hypothetical) new POCTs
127	van Hecke, O.; Butler, C.; Mendelson, M.; Tonkin-Crine, S.	2015	Journal of the International AIDS Society	What are the constraints and opportunities for HIVST scale-up in Africa? Evidence from Kenya, Malawi and South Africa	To assess the perceptions of HIV self-testing among stakeholders in Kenya, Malawi and South Africa from the perspectives of government policy makers, academics, activists, donors, procurement specialists, laboratory practitioners and health providers
128	van Rooyen, H.; Tulloch, O.; Mukoma, W.; Makusha, T.; Chepuka, L.; Knight, L. C.; Peck, R. B.; Lim, J. M.; Muturi, N.; Chirwa, E.; Taegtmeyer, M.	2022	Diagnostics	Can Self-Administered Rapid Antigen Tests (RATs) Help Rural India? An Evaluation of the CoviSelf Kit as a Response to the 2019-2022 COVID-19 Pandemic	To evaluate the practicality of a self-administered rapid antigen test kit against COVID-19 in rural India on the basis of data in its instructional leaflet, reports about India's 'digital divide', and published research on the constraints of daily life in Indian villages
129	Vicziany, M.; Hardikar, J.	2019	Aids and Behavior	"Closing the Gap": Provider Recommendations for Implementing Birth Point of Care HIV Testing	To describe Kenyan providers' recommendations for optimal implementation of birth POC testing for HIV, as pragmatic guidance for the introduction of infant POC testing in Kenya and similar settings.
130	Wexler, C.; Kamau, Y.; Halder, R.; Brown, M.; Maloba, M.; Mabachi, N.; Sandbulte, M.; Gautney, B.; Goggin, K.; Odeny, T.; Finocchario-Kessler, S.	2019	PLoS ONE	Factors affecting acceptance of at-birth point of care HIV testing among providers and parents in Kenya: A qualitative study	To evaluate factors influencing the provision and acceptance of at-birth POC testing from the perspectives of both HIV care providers and parents of HIV-exposed infants in Kenya

131	Wexler, C.; Maloba, M.; Brown, M.; Mabachi, N.; Goggin, K.; Gautney, B.; Odeny, B.; Finocchario-Kessler, S.	2011	Malaria Journal	Exploring provider and community responses to the new malaria diagnostic and treatment regime in Solomon Islands	To understand community and prescriber perceptions and acceptability of the new diagnostic and treatment interventions for malaria in Solomon Islands, which include the introduction of rapid diagnostic testing, from the perspectives of community members, healthcare workers and community leaders
132	Wijesinghe, R. S.; Atkinson, J. A. M.; Bobogare, A.; Wini, L.; Whittaker, M.	2016	Global Public Health	Barriers and facilitators to voluntary HIV testing uptake among communities at high risk of HIV exposure in Chennai, India	To explore multi-level barriers and facilitators to HIV testing, and experiences with free, publicly available testing services from the perspectives of men who have sex with men, transgender women, cisgender female sex workers, and injecting drug users in India
133	Woodford, M. R.; Chakrapani, V.; Newman, P. A.; Shunmugam, M.	2021	BMC Health Services Research	Operational experiences associated with the implementation of near point-of-care early infant diagnosis of HIV in Myanmar: a qualitative study	To explore and document the challenges and enabling factors in implementing the POC Xpert® HIV-1 Qual test for early infant diagnosis as part of routine services in four public hospitals in Myanmar, from the perspectives of caregivers, healthcare providers, and key informants.
134	Yee, W. L.; Hla, H.; Mohamed, Y.; Nightingale, C. E.; Htay Htay, T.; Win, T.; Latt Latt, K.; Win Win, Y.; Moe Myat, A.; Badman, S. G.; Vallely, A. J.; Anderson, D.; Kelly-Hanku, A.; Luchters, S.	2020	PLoS ONE	Caregiver experience and perceived acceptability of a novel near point-of-care early infant HIV diagnostic test among caregivers enrolled in the PMTCT program, Myanmar: a qualitative study	To explore experiences of caregivers of HIV-exposed infants enrolled in the prevention of mother-to-child transmission of HIV (PMTCT) program in Myanmar and the perceived acceptability of point-of-care early infant diagnosis testing compared to conventional centralised laboratory-based testing.
135	Yee, W. L.; Than, K. K.; Mohamed, Y.; Htay, H.; Tin, H. H.; Thein, W.; Kyaw, L. L.; Yee, W. W.; Aye, M. M.; Badman, S. G.; Vallely, A. J.; Luchters, S.; Kelly-Hanku, A.	2019	BMC Health Services Research	Integrated point-of-care testing (POCT) for HIV, syphilis, malaria and anaemia at antenatal facilities in western Kenya: a qualitative study exploring end-users' perspectives of appropriateness, acceptability and feasibility	To capture experiences of using POCTs for HIV, syphilis, malaria and anaemia, and to assess the appropriateness, acceptability and feasibility of integrated testing for antenatal care in Kenya from the perspective of healthcare workers
136	Young, N.; Achieng, F.; Desai, M.; Phillips-Howard, P.; Hill, J.; Aol, G.; Bigogo, G.; Laserson, K.; Kuile, F. ter; Taegtmeyer, M.	2016	Physis Revista de Saúde Coletiva	Descentralização da atenção em HIV-Aids para a atenção básica: tensões e potencialidades	To analyse the tensions within the process of decentralisation of HIV and syphilis testing and follow-up of PLWHA
137	Zambenedetti, G.; Silva, R.A.N. da.	2018	SAGE Open Medicine	Dengue rapid diagnostic tests: Health professionals' practices and challenges in Burkina Faso	To analyse the use of rapid diagnostic tests for dengue fever after their introduction in six health and social promotion centres in Burkina Faso, from the perspective of health professionals

138	Zongo, S.; Carabali, M.; Munoz, M.; Ridde, V.	2016	Malaria Journal	A qualitative study of health professionals' uptake and perceptions of malaria rapid diagnostic tests in Burkina Faso	To learn about the adoption of rapid diagnostic tests (RDT) for malaria in the natural context of a national scale-up policy in Burkina Faso from the perspectives of health professionals and members of the health district management teams
